# A Kinetically Superior Rechargeable Zinc‐Air Battery Derived from Efficient Electroseparation of Zinc, Lead, and Copper in Concentrated Solutions

**DOI:** 10.1002/cssc.202200039

**Published:** 2022-04-20

**Authors:** Peng Chen, Xia Wang, Dongqi Li, Tobias Pietsch, Michael Ruck

**Affiliations:** ^1^ Faculty of Chemistry and Food Chemistry Technische Universität Dresden 01062 Dresden Germany; ^2^ Max Planck Institute for Chemical Physics of Solids 01187 Dresden Germany

**Keywords:** batteries, kinetics, electroseparation, solvent effects, zinc

## Abstract

Zinc electrodeposition is currently a hot topic because of its widespread use in rechargeable zinc‐air batteries. However, Zn deposition has received little attention in organic solvents with much higher ionic conductivity and current efficiency. In this study, a Zn‐betaine complex is synthesized by using ZnO and betainium bis[(trifluoromethyl)sulfonyl]imide and its electrochemical behavior for six organic solvents and electrodeposited morphology are studied. Acetonitrile allowed dendrite‐free Zn electrodeposition at room temperature with current efficiencies of up to 86 %. From acetonitrile solutions in which Zn, Pb, and Cu complexes are dissolved in high concentrations, Zn and Pb/Cu are efficiently separated electrolytically under potentiostatic control, allowing the purification of solutions prepared directly from natural ores. Additionally, a highly flexible Zn anode with excellent kinetics is obtained by using a carbon fabric substrate. A rechargeable zinc‐air battery with these electrodes shows an open‐circuit voltage of 1.63 V, is stable for at least 75 cycles at 0.5 mA cm^−2^ or 33 cycles at 20 mA cm^−2^, and allows intermediate cycling at 100 mA cm^−2^.

## Graphical abstract

**That zinc‐ing feeling**: Electrodeposition in organic solvents allows efficient separation of Zn from Pb and Cu in concentrated solutions of their betaine complexes. This method is used to prepare Zn‐coated carbon‐fabric electrodes that exhibit superior kinetics and inhibition of dendrite formation. A rechargeable zinc‐air battery with this anode has an open‐circuit voltage of 1.63 V and excellent cycling stability and rate performance.

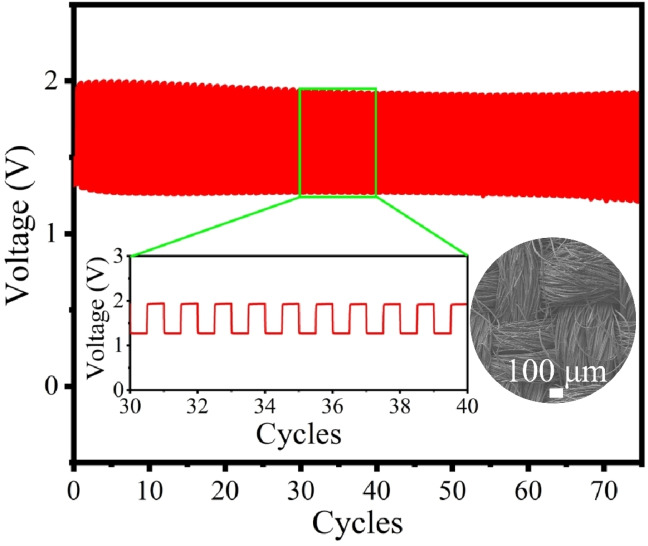

## Introduction

### Ionic liquids for metal processing

Metals are widely used in daily life, energy storage and many other areas,^[1]^ making their extraction from natural resources and their recovery from waste of high significance to industry and the circular economy.[^2^, ^3^] By using metal oxides or sulfide sources, conventional metallurgical processes typically consume large amounts of energy, emit a lot of CO_2_ due to the high temperatures required, and produce large amounts of often problematic waste.[^3^, ^4^, ^5^] The acceleration of climate change and the increasing scarcity of non‐renewable energy sources require more energy‐efficient, economical and environmentally friendly methods of extracting metal oxides from natural resources or industrial waste and the subsequent production of metals and their compounds.[^6^, ^7^] Ionic liquids (ILs) provide a new type of reaction environment that combines a number of very useful properties.^[8]^ An IL is a salt that is in the liquid state at room temperature (RT) or at least below 100 °C.[^3^, ^9^] They usually have high ionic conductivity,^[10]^ low vapor pressure, are non‐flammable^[11]^ and have relatively low toxicity.[^12^, ^13^, ^14^] By exchanging the organic moieties, the properties of ILs can be adjusted to suit a wide range of applications.[^9^, ^13^]

At present, two main types of ILs are used for electrodeposition: first‐generation chloridoaluminate ILs, and second‐generation “air‐ and water‐stable” ILs with anions such as tetrafluoroborate, (BF_4_^−^), hexafluorophosphate (PF_6_^−^), trifluoromethanesulfonate ([OTf]^−^, CF_3_SO_3_^−^) or bis(trifluoromethylsulfonyl)imide ([TFSI]^−^, [NTf_2_]^−^, (CF_3_SO_2_)_2_N^−^).[^1^, ^3^, ^9^] A large number of studies have been reported on the electrodeposition of single metals or alloys based on chloridoaluminate ILs.[^15^, ^16^, ^17^, ^18^] The biggest drawback of first‐generation ILs is that they are extremely susceptible to hydrolysis and require inert conditions for electrodeposition.^[3]^ Therefore, replacing the water‐sensitive anion in first‐generation ILs is an important step towards practical applications.[^11^, ^19^, ^20^] Most importantly, many ILs have an electrochemical window wide enough to deposit metals that cannot be deposited from aqueous solutions.[^3^, ^13^] In addition, the emerging ILs analogue, deep eutectic solvents (DESs), is also being used as an electrolyte for metal electrodeposition.^[21]^

The second‐generation IL betainium bis[(trifluoromethyl)sulfonyl]imide ([Hbet][NTf_2_]; [Hbet]^+^=betainium, (CH_3_)_3_N^(+)^CH_2_COOH)), is particularly suited for this process owing to its redox‐stability and specific‐functionalization.^[7]^ Betaine, which constitutes the cationic part of this task‐specific IL (TSIL), is a cheap, abundant, and biodegradable byproduct of sucrose processing from sugar beets.^[3]^ It has been demonstrated that [Hbet][NTf_2_] is able to dissolve many metal oxides, especially in the presence of water or chloride.[^1^, ^3^, ^7^] The interaction of [Hbet][NTf_2_] with the metal cation occurs through the carboxyl group of the betainium cation. In a typical metal oxide dissolution process [Eq. (1)], the acidic protons of the carboxyl groups react with the oxide ions of the metal oxide to H_2_O, which is evaporated at the applied temperature of about 175 °C, and in parallel the now deprotonated carboxylate groups coordinate the metal cations.^[3]^

(1) $$MO+2\left[\mathrm{HBet}\right]\left[\mathrm{NTf}{}_{2}\right]\to \left[M\left(\mathrm{bet}\right){}_{2}\right]\left[\mathrm{NTf}{}_{2}\right]{}_{2}+H{}_{2}O$$

Due to these useful properties, research on the solubility of metal oxides^[7]^ and the extraction of elements^[22]^ in [Hbet][NTf_2_] are in great progress. A remaining task is to replace the anion for a perspective application on a larger scale, since [NTf_2_]^−^ is expensive and not environmentally friendly.

Compared to lithium‐ion batteries, Zn batteries are considered as the next generation of anode alternatives due to their high theoretical capacity (820 mAh g^−1^), abundance,^[23]^ environmental impact (nontoxicity)[^24^, ^25^] and compatibility with water or organic electrolytes,[^24^, ^25^] driving the boom of Zn battery research.[^26^, ^27^] In this context, the research on the electrodeposition of Zn has been a hot topic in recent years.

The electrolytic processing of Zn is very sensitive to impurities.^[28]^ The most common Zn ores are sphalerite and wurtzite (zinc sulfide, ZnS), smithsonite (zinc carbonate, ZnCO_3_) and hemimorphite (Zn_4_(OH)_2_[Si_2_O_7_]⋅H_2_O).[^3^, ^29^] About 40 % of the worldwide Zn metal is produced by the electrolysis of zinc sulfate (ZnSO_4_).[^3^, ^29^] For this purpose, zinc ore is converted into zinc oxide (ZnO) by a series of high‐temperature procedures and then leached with sulfuric acid (H_2_SO_4_) to produce a ZnSO_4_ solution.[^30^, ^31^] Zn ores typically contain also lead (galena, PbS), iron (pyrite or marcasite, FeS_2_), and copper minerals (chalcopyrite, CuFeS_2_), which requires an efficient separation process before Zn can be used in a battery.

There has been a lot of research on the Zn electrodeposition from alkaline aqueous environments.^[32]^ On the one hand, the use of strong acids and bases places higher demands on the equipment and its maintenance. In water‐based electrolytes, on the other hand, Zn electrodeposition from aqueous solutions is mainly accompanied by the decomposition of water, as the standard electrode potential of Zn (−0.76 V vs. standard hydrogen electrode, SHE) is more negative than that of the hydrogen electrode (−0.83 V vs. SHE). As a result, even under optimal conditions, energy efficiencies of only about 60 to 70 % are achieved.^[13]^

In the search for alternatives, Zn has been successfully electrodeposited from chloridoaluminate ILs,[^15^, ^16^, ^33^, ^34^, ^35^] some air‐ and water‐stable ILs,[^3^, ^29^] and choline chloride (ChCl)/ethylene glycol (EG) DES.[^36^, ^37^] The high viscosity of ILs and DESs, however, greatly inhibits ion mobility and therefore the current efficiency of the Zn electrodeposition is unfavorable.[^3^, ^38^] Consequently, it will be particularly important to improve this dilemma, for example, by using organic solvent alternatives that not only provide high current efficiency but also achieve high ionic conductivity.^[38]^

### Rechargeable zinc‐air batteries

**Scheme 1:**
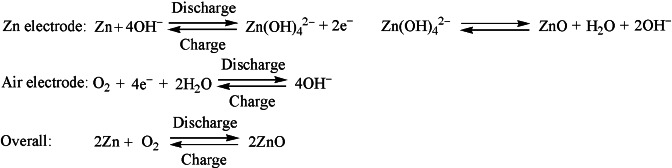
The charge‐discharge reaction mechanism for ZABs.

Rechargeable zinc‐air batteries (ZABs) are widely considered as one of the most promising energy storage devices, owing to their high specific energy density (1084 Wh kg^−1^), unique semi‐closed systems, low cost, and environmentally friendly nature (Scheme 1).[^39^, ^40^]

There are two main avenues for the development of ZABs: electrochemical cells based on either aqueous alkaline or aprotic electrolytes.^[41]^ For water‐based electrolytes, their low energy efficiency is a fundamental problem.^[13]^ In addition, the volatility of the electrolyte, corrosion, the appearance of carbonate passivation layers (because alkaline aqueous solutions are very sensitive to atmospheric CO_2_), and especially the formation of Zn dendrites (see below) limit the further development of ZABs.^[13]^

On the cathode side of ZABs, the oxygen evolution reaction (OER) and the oxygen reduction reaction (ORR) strongly influence the charge‐discharge kinetics and energy efficiency of ZABs. Great progress has been made in the development of bifunctional electrocatalysts for these processes.[^39^, ^40^, ^42^, ^43^, ^44^, ^45^, ^46^]

Moreover, fundamental problems on the Zn anode side must be addressed.^[47]^ Firstly, the corrosion of the Zn anode is a parasitic reaction, in which Zn reacts with water to produce zinc hydroxide (Zn(OH)_2_) or, in alkaline solution, [Zn(OH)_4_]^2−^ and hydrogen gas. Both, the hydroxide and the tetrahydroxozincate, decompose into ZnO, which passivates the Zn electrode because of its low electrical conductivity and reduces the contact surface area of the Zn electrode with the electrolyte.^[47]^ The result is a loss of capacity. Secondly, during the charging process, dendritic Zn is produced on the surface of the anode and can penetrate the diaphragm between the cathode and anode, leading to short circuits.^[47]^

In recent years, there are reports to improve cycling stability by adding additives (such as aluminum oxide, bismuth oxide),[^47^, ^48^] surface coating or plating with bismuth‐based oxide glass for Zn particle anodes,^[49]^ and high surface area Zn anodes^[47]^ to inhibit to some extent self‐discharge introduced by corrosion or dendrite formation.^[47]^ Besides, Zn anodes with not only a large specific surface area but also a reduced Zn deposition overpotential can play the triple role of simultaneously increasing activity, effectively suppressing passivation‐induced performance degradation, and inhibiting dendrite formation.^[47]^ This is one approach we take in this study.

Secondly, to proceed in the development of fast electrodeposition using organic solvents we studied the electrodeposition of Zn, Pb, and Cu from solutions of their betaine‐based complexes in six different solvents, including acetonitrile (AN), acetone, *N*,*N*‐dimethylformamide (DMF), methanol, propylene carbonate (PC) and sulfolane. We were interested in the chemical and electrochemical behavior of those solutions under the conditions of electrodeposition, the effect of solvents on the morphology of the product layers, and options for sequential deposition of Zn and Pb from mixtures to investigate the possibilities of separation of impurities introduced by the natural resources used.

Eventually, we combined electrodes with large specific surface area and an aprotic electrolyte. We tested the performance of a Zn layer deposited from AN solution on three‐dimensional Cu foam (^3D^Cu) as Zn/^3D^Cu anode and 3D carbon fabric (^3D^CF) as Zn/^3D^CF anode in symmetric Zn batteries with organic electrolytes as well as in rechargeable ZABs.

## Results and Discussion

### Comparison of Zn electrodeposition in six organic solvents

**Figure 1:**
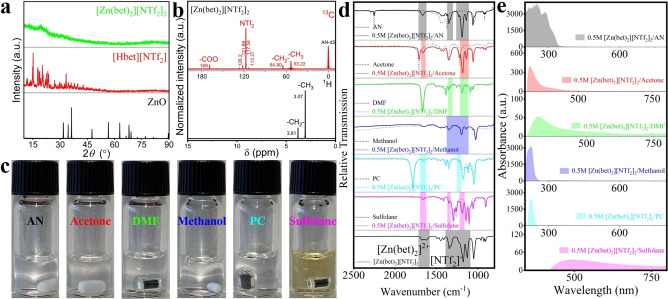
Chemical characterization of [Zn(bet)_2_][NTf_2_]_2_. (a) Comparison of PXRD patterns with pure [Hbet][NTf_2_] and ZnO. (b) ^1^H and ^13^C NMR spectra. (c) Solubility photographs in six different organic solvents at 0.5 m. (d,e) Comparison of six different electrolytes at 0.5 m: (d) FTIR spectra in the range of 800 cm^−1^≤*ῦ*≤2500 cm^−1^; (e) UV/Vis absorption spectra.

The maximum molar ratio of *n*_ZnO_/*n*_IL_=1 : 2, in which ZnO can be completely dissolved, is a first hint that the expected [Zn(bet)_2_]^2+^ complex with tetrahedrally coordinated Zn^2+^ cations formed. The [Zn(bet)_2_][NTf_2_]_2_ looks like colorless and transparent glass that can be broken by hand (see the Supporting Information, Figure S1). There is a big difference of the PXRD patterns between [Zn(bet)_2_][NTf_2_]_2_ and the pure [Hbet][NTf_2_] and ZnO (Figure 1a), which shows the amorphous structure of solid [Zn(bet)_2_][NTf_2_]_2_.^[50]^ Figure 1b shows the liquid‐phase ^13^C NMR spectrum of [Zn(bet)_2_][NTf_2_]_2_, which showed no additional or absent signals compared to the pure IL [Hbet][NTf_2_],^[3]^ but the resonances were slightly shifted upfield. As in our previous study,[^3^, ^8^] we explain this by the deprotonation of the carboxyl group and the formation of the complex [Zn(bet)_2_]^2+^, which reduces the electron abstraction from the C atom and increases the shielding of the nuclei.[^3^, ^8^, ^51^] Another important piece of evidence for coordination Zn^2+^ cations of [Zn(bet)_2_]^2+^ complex is that the signal in the ^1^H NMR spectrum that originally belonged to the carboxyl group at about 8.30 ppm completely disappeared (Figure 1b).^[3]^

The synthesized [Zn(bet)_2_][NTf_2_]_2_ glass was dissolved in six different solvents, AN, acetone, DMF, methanol, PC and sulfolane. At least up to 0.5 m, [Zn(bet)_2_][NTf_2_]_2_ dissolved completely in all solvents, resulting in clear and transparent solutions (Figure 1c). In AN, solubility up to 4.5 m was observed. All solutions are colorless except for sulfolane, which is a yellowish liquid. The fastest dissolution was observed in AN, which illustrates that highly polar organic solutions are favorable for the dissolution of [Zn(bet)_2_][NTf_2_]_2_.^[50]^ Before we evaluated the electrochemical properties of the solution, we determined the IR and UV/Vis spectra of these six different electrolytes compare to the pure [Zn(bet)_2_][NTf_2_]_2_. Except DMF based electrolytes, the highlighted coordination of the betaine can be clearly seen by comparing the IR spectra of the pure [Zn(bet)_2_][NTf_2_]_2_ and different electrolytes (Figure 1d; enlarged images in Figure S2). The [Hbet]^+^ cation is characterized by the asymmetric stretching vibrational absorption bands of the carboxyl OH (*υ*_as_(OH)=3249 cm^−1^) and COO (*υ*_as_(COO)=1756 cm^−1^).^[3]^ For the [Zn(bet)_2_][NTf_2_]_2_, a replaced absorption band from 1571 cm^−1^ to 1722 cm^−1^ was observed (Figure 1d), which we attribute to the vibration of carboxylate groups coordinated to Zn^2+^. And from 1095 cm^−1^ to 1376 cm^−1^ is another highlighted [NTf_2_]^−^ signals (Figure 1d). Whereas for DMF based electrolytes, the coordination invisibility of the [Zn(bet)_2_]^2+^ complex due to the DMF solvent at 1662 cm^−1^ with intrinsic asymmetric stretching vibrational absorption bands. Accordingly, the UV/Vis spectra of these different electrolytes showed absorption bands between 200 and 600 nm, which can be attributed to charge transfer transitions in the [Zn(bet)_2_]^2+^ complex (Figure 1e). The difference in peak intensity is caused by the distinction in polarity and pH of the solvents.^[52]^

**Figure 2:**
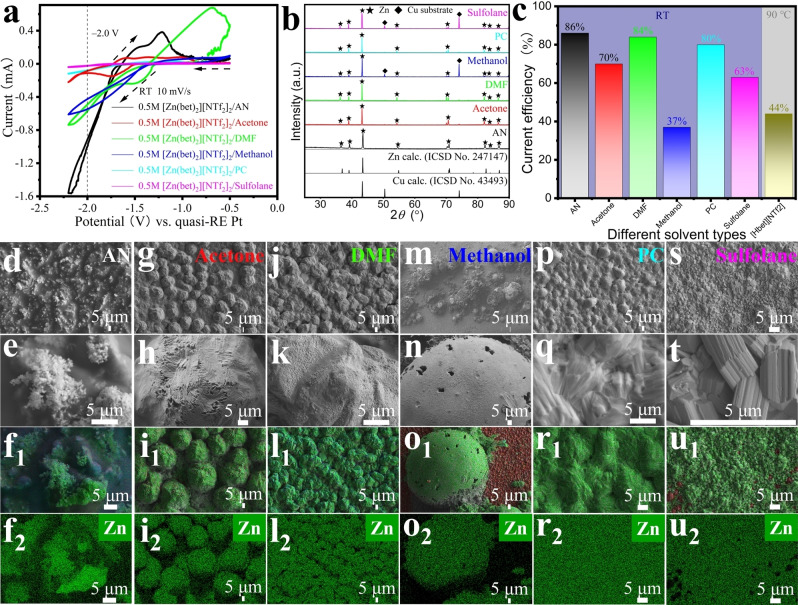
(a) The CV of the six different 0.5 m electrolytes at a scan rate of 10 mV s^−1^ and RT; the electrodeposition potential is indicated with dotted line (The arrows point to the start and direction of the potential scan). Characterization of the Zn layer that was electrodeposited at −2.0 V and RT on Cu foil within 5 h from six different electrolytes. (b) The PXRD patterns comparison of electrodeposited Zn layer. (c) Current efficiency comparison in six different organic solvents at RT or IL [Hbet][NTf_2_] system at 90 °C reported in Ref. [3]. (d–u) SEM images and EDX mappings of all elements of the Zn distribution of the Zn layer electrodeposited from AN (d,e,f_1_,f_2_), acetone (g,h,i_1_,i_2_), DMF (j,k,l_1_,l_2_), methanol (m,n,o_1_,o_2_), PC (p,q,r_1_,r_2_), or sulfolane (s,t,u_1_,u_2_).

To electrodeposit Zn from these six different electrolytes by controlled potential coulometry technique, we used a three‐electrode electrolytic cell. The working, counter and reference electrodes were Cu foil, a Pt wire and a Pt plate, respectively. Detailed CV investigation of the solutions (Figure 2a) revealed that the distinctive reduction of Zn^2+^ to Zn starts at approximately −1.64 V (AN), −1.62 V (PC), −1.68 V (sulfolane) (Figure S3a, e, f and Figure S4a, e, f).^[3]^ Moreover, since organic solvents acetone and DMF have respectively reductive decomposition reactions at potentials −1.40 V and −1.10 V (Figure S3b, c), making the CV curves appear first at potentials −1.44 V and −1.24 V for the reductive decomposition reactions of organic solvents, and then only at −1.74 V and −1.64 V for the electroreduction of Zn^2+^ to Zn (Figure S4b, c). The most difficult to distinguish is methanol based solutions, which has a reductive decomposition reaction at potential −1.34 V (Figure S3d), making the CV curve appear from potential −1.32 V with the reductive decomposition reaction of the organic solvent, and then it is impossible to distinguish the potential of the electroreduction of Zn^2+^ to Zn (Figure S4d). The peak potential of the reverse oxidation process also varies greatly in different solvent system. Overall, there is only a small difference in the potential of electroreduction of Zn^2+^ to Zn, so different solvents do not noticeably affect the potential of Zn^2+^ electroreduction. Consequently, as marked by the dashed line (Figure 2a), −2.0 V (between working and reference electrode) was chosen as the reduction potential in order to compare the effect of different solvents on the morphology of Zn electrodeposition. During the electrooxidation of the solutions at the anode, we did not observe anything at the counter electrode, but an odor and some color changes of the solutions.

The electrodeposition of Zn is quite uniform (Figure 2j,k,p,q,s,t) and covers the entire Cu substrate (Figure S5) from DMF‐, PC‐, and sulfolane‐based solutions. The depositions are all at the micron scale, in DMF (other magnification, Figure S6) and PC (other magnification, Figure S7), in the form of rounded mound‐like protrusions at adjacent edges with faintly discernible layers of microcrystalline overlays (Figure S6a and Figure S7c, d), about 20 to 30 μm, and in sulfolane (other magnification, Figure S8), in the form of very homogeneous and finer layered stacked crystal structures, about 2 to 3 μm. The EDX image of electrodeposited Zn and the corresponding Zn elemental distribution from the EDX signals (Figures S9, S10 and S11and Figure 2l_1_,l_2_,r_1_,r_2_,u_1_,u_2_) are in accordance with the results discussed above. Analogous to the experiments in DMF or PC, in acetone, the Zn morphology remains as a tent‐like morphology of 40–50 μm (Figure 2g,h), and a morphology of 2 to 3 μm layered microcrystalline stacks can be distinguished (Figure S12). In conjunction with this, the EDX image and the corresponding Zn elemental distribution from the EDX signals (Figure S13) are shown in Figure 2i_1_,i_2_. In contrast, the morphology of electrodeposited Zn in methanol is very different. Although there are still rounded mound‐like protruding morphologies of about 100 to 150 μm (Figure 2m,n), the majority of them are irregular skeleton‐like cross structures, and the mound‐like Zn deposits are not dense internally but are supported by a skeleton structure (Figure S14). The EDX image and the corresponding Zn elemental distribution from the EDX signals (Figure S15) are shown in Figure 2o_1_,o_2_. In AN, we obtained microspherical Zn particles of about 1 μm (Figure 2d,e) agglomerated into larger particles (Figure S16). Furthermore, the EDX image and the corresponding Zn elemental distribution from the EDX signals (Figure S17) are shown in Figure 2f_1_,f_2_. The PXRD indicated high crystallinity of the electrodeposited Zn from all six different solutions (Figure 2b). Before and after 5 h Zn electrodeposition experiments, the changes in the chemical properties of the solutions were also explored in order to learn about the electrooxidation reactions. However, based on the IR (Figure S18), no significant changes were observed, and it is speculated that this may be due to the large amount of remaining [Zn(bet)_2_]^2+^ in the solution.^[3]^ For the UV/Vis spectra (Figure S19) after electrodeposition, the positions of the main peaks were all still at about 200 and 600 nm, which was also due to the large amount of remaining [Zn(bet)_2_]^2+^ in the solution. In all the solvents except sulfolane, the main peak evolves more broadly and the intensity of the peaks increased, probably due to the electrooxidation products and the decomposition of the solvent.

As shown by the CV data (Figure 2a), the AN solution has the largest current value at the same potential compared to other solvent systems, indicating that the lowest solution viscosity gives the largest ionic conductivity.^[53]^ This implies a strong influence of solution viscosity on cation transport and, consequently, on nucleation and growth of the product layer morphology.[^51^, ^54^] The higher the ionic conductivity of the solution, the faster the cation transport, the smaller the grains and the denser the layer.[^3^, ^53^] Correspondingly, the maximum current efficiency at RT is obtained in the AN system, 86 %, compared to 84 % in DMF or 80 % in PC (Figure 2c). In the IL [Hbet][NTf_2_] system reported in Ref. [3], a current efficiency of only 44 % had been achieved, and this only when heated to 90 °C (Figure 2c).^[3]^ It is also worth mentioning that the melting point of pure [Hbet][NTf_2_] is 54 °C.^[3]^ Therefore, electrodeposition of Zn at RT is not possible, whereas in AN, fast, dense and uniform Zn electrodeposition can be obtained at RT and under atmospheric conditions.

In conclusion, Zn electrodeposition can be obtained in all six solvents, indicating that this room temperature and high current efficiency organic solvents‐based electrodeposition method has great industrial practical application value compared to aqueous and IL systems, and has the prospect of large‐scale application. The most uniform Zn electrodeposition morphology was obtained in sulfolane, but in AN, not only smaller microcrystalline deposits could be obtained, but also the highest current efficiency, so AN was chosen for follow‐up experiments.

### Efficient electroseparation of Zn and Pb, or Zn and Pb/Cu in concentrated AN solutions

We have demonstrated that Zn can be electrodeposited individually from [Zn(bet)_2_][NTf_2_]_2_ solutions, prepared from ZnO, in six different solvents at RT, in an environmentally friendly, resource‐saving manner. However, natural ores, earths or minerals are mixtures of various metal‐containing compounds and concomitant matter.[^1^, ^3^, ^7^] If [Zn(bet)_2_][NTf_2_]_2_ is to be synthesized using an unrefined ZnO source, dissolved impurity metals must be separated to avoid contamination of the deposited Zn. Such separation can be achieved, for example, by selective leaching of the feedstock and/or by stepwise electrodeposition. In the latter case in particular, a highly concentrated solution must be used to meet industrial requirements. In ore deposits, Zn is usually accompanied by Pb and Cu. Therefore, we tested solutions containing Zn and Pb or all three of these elements for achievable efficiency in electroseparation at high concentrations.

**Figure 3:**
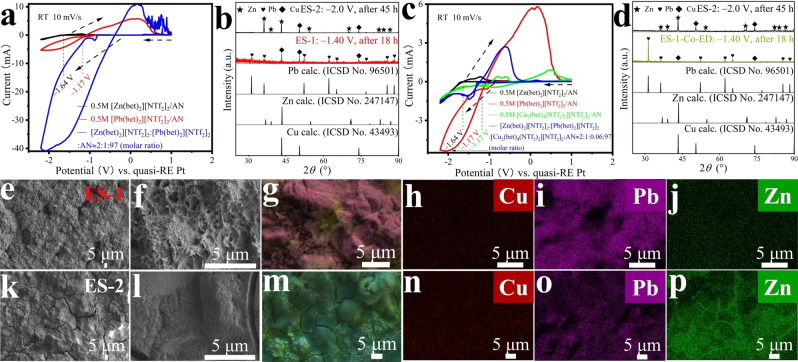
Electroseparation of Zn from Pb (a,b,e–p) and Zn from Pb/Cu (c,d). (a) CV comparison of solutions of pure 0.5 m [Zn(bet)_2_][NTf_2_]_2_, [Pb(bet)_2_][NTf_2_]_2_, and mixture in AN (*n* ${}_{\mathrm{Zn}\left(\mathrm{bet}\right){}_{2}]\left[\mathrm{NTf}{}_{2}\right]{}_{2}}$ /*n* ${}_{\left[\mathrm{Pb}\left(\mathrm{bet}\right){}_{2}\right]\left[\mathrm{NTf}{}_{2}\right]{}_{2}}$ /*n*_AN_≈2 : 1 : 97) at a scan rate of 10 mV s^−1^ and RT; the initial electroreduction potentials are indicated with dotted lines. (b,d) Comparison of the PXRD pattern of the coated Cu electrodes with reference patterns at different state. (c) CV comparison of solutions of pure 0.5 m [Zn(bet)_2_][NTf_2_]_2_, [Pb(bet)_2_][NTf_2_]_2_, [Cu_2_(bet)_4_(NTf_2_)_2_][NTf_2_]_2_ and mixture in AN (*n* ${}_{\mathrm{Zn}\left(\mathrm{bet}\right){}_{2}]\left[\mathrm{NTf}{}_{2}\right]{}_{2}}$ /*n* ${}_{\left[\mathrm{Pb}\left(\mathrm{bet}\right){}_{2}\right]\left[\mathrm{NTf}{}_{2}\right]{}_{2}}$ /*n* ${}_{\left[\mathrm{Cu}{}_{2}\left(\mathrm{bet}\right){}_{4}\left(\mathrm{NTf}{}_{2}\right){}_{2}\right]\left[\mathrm{NTf}{}_{2}\right]{}_{2}}$ /*n*_AN_≈2 : 1 : 0.06 : 97) at a scan rate of 10 mV s^−1^ and RT. (e–p) SEM images and EDX elemental mappings of the Cu, Pb and Zn distribution of first step (e–j) and second step (k–p).

We started with [Zn(bet)_2_][NTf_2_]_2_ and [Pb(bet)_2_][NTf_2_]_2_ dissolved in AN (*n* ${}_{\mathrm{Zn}\left(\mathrm{bet}\right){}_{2}]\left[\mathrm{NTf}{}_{2}\right]{}_{2}}$ /*n* ${}_{\left[\mathrm{Pb}\left(\mathrm{bet}\right){}_{2}\right]\left[\mathrm{NTf}{}_{2}\right]{}_{2}}$ /*n*_AN_≈2 : 1 : 97). White, nearly transparent [Pb(bet)_2_][NTf_2_]_2_ (Figure S20) can be made from PbO and [Hbet][NTf_2_] (*n*_PbO_/*n*_IL_=1 : 2) by using the same approach as described above for ZnO. In the CV data for the [Pb(bet)_2_][NTf_2_]_2_/AN solution, marked by the red dashed line, the Pb^2+^ complex has an electroreduction potential to Pb of approximately −1.17 V (Figure 3a). From the [Pb(bet)_2_][NTf_2_]_2_/AN solution, a black uniform and bulk Pb electrodeposition (Figure S21) is obtained by setting the voltage to −1.17 V in a constant potential process, which is consistent with the initial electroreduction potential of the Pb^2+^ complex as read in the CV data (Figure 3a). The peak potential of the reverse oxidation process is about 0.0 V.

The electroreduction potential of the Zn^2+^ complex within 0.5 m [Zn(bet)_2_][NTf_2_]_2_/AN solution is approximately −1.64 V (Figure 3a). Thus, the pronounced difference in the initial electroreduction potentials of the Zn^2+^ and Pb^2+^ complexes enables efficient separation of Zn and Pb from a mixed solution. As conjectured, the most obvious difference in CV is that the electroreduction potential of the mixed solution is pulled to about −1.17 V due to the presence of Pb(bet)_2_][NTf_2_]_2_ (Figure 3a). The electroreduction peaks of the two cations constitute a fusion into one, making the electroreduction peak of Zn^2+^ no longer distinguishable, but it is believed that the electroreduction potential of Zn^2+^ is still about −1.64 V (other concentration, Figure S22a).^[3]^ Furthermore, for the noise in the CV of Figure 3a, if impurities are reactive, they can have an enormous effect on the CV, so we suspect that it is due to the presence of impurities.

Applying −1.40 V in the controlled potential coulometry setup to obtain fast nucleation, Pb was deposited on a Cu substrate at RT for about 18 h from a stirred bimetallic solution, with the aim of reducing as much of the Pb^2+^ as possible (Figure S22b). After 18 h of Pb electrodeposition, the solution had changed from colorless to a light brown (Figure S22b). Based on the weight of the electrodeposited Pb metal, only 1.5 % of the [Pb(bet)_2_]^2+^ complex remained in solution (Figure S22c). According to the PXRD pattern, the deposited layer on the Cu substrate consisted only of Pb, without any traces of Zn (Figure 3b). The Pb layer is rather dense with some small hollows (Figure 3e,f and Figure S23), which corresponds to the rapid nucleation at high overpotential. The fast deposition, which was induced by the high overpotential, promoted nucleation over crystal growth.[^51^, ^54^, ^55^] Very small traces of Zn detected in EDX mappings (Figure 3g–j and Figure S24) are probably due to solvent residues.

Continuously, after replacing the working electrode with a new Cu foil, the second step of the electroseparation was carried out at the increased potential of −2.0 V for about 27 h. The aim was to get the Zn from solution. With the continued reaction, the color of the solution continued to deepen and changed to dark brown (Figure S22b). At this point, about 41 % of the active [Zn(bet)_2_]^2+^ complex remained in solution (Figure S22c). The PXRD of the deposited material showed only Zn (Figure 3b), but traces of lead could be detected in high‐resolution EDX mappings (Figure 3m–p and Figure S25). Although the Zn morphology is still dense with faintly visible layered microcrystals, similar to the dense morphology obtained from simple solutions (Figure 2d), cracks due to crystal nucleation and rapid growth were present (Figure 3k,l and Figure S26).

For a more advanced separation task, we additionally dissolved CuO to create a trimetallic mixture with the molar ratio *n* ${}_{\mathrm{Zn}\left(\mathrm{bet}\right){}_{2}]\left[\mathrm{NTf}{}_{2}\right]{}_{2}}$ /*n* ${}_{\left[\mathrm{Pb}\left(\mathrm{bet}\right){}_{2}\right]\left[\mathrm{NTf}{}_{2}\right]{}_{2}}$ /*n* ${}_{\left[\mathrm{Cu}{}_{2}\left(\mathrm{bet}\right){}_{4}\left(\mathrm{NTf}{}_{2}\right){}_{2}\right]\left[\mathrm{NTf}{}_{2}\right]{}_{2}}$ /*n*_AN_≈2 : 1 : 0.06 : 97. This solution of ZnO, PbO and CuO in [Hbet][NTf_2_] yielded also a brittle solid at RT (Figure S27b). More encouragingly, blue [Cu_2_(bet)_4_(NTf_2_)_2_][NTf_2_]_2_ (Figure S27a) can also be dissolved rapidly at RT and in similar concentration in AN. Thus, after dissolving in AN and removing unreacted CuO by centrifugation, a clear blue solution containing the three different metal complexes was obtained (Figure S27c,d).

The potential for electroreduction of Cu^2+^ to metallic Cu was difficult to determine from the CV data, which could be attributed to the intermediate formation of Cu^+^ (green curve in Figure 3c). Using the constant potential technique, at −1.1 V and −1.15 V, the electrooxidation reaction occurs, which the Cu foil working electrode dissolved in the solution (Cu to Cu^+^ or Cu^2+^) and only at −1.17 V Cu was electrodeposited (Figure S28). Addition of chloride ions during preparation of the precursor could also be a reason for the multiple redox peaks in the CV curve containing Cu‐betaine complex. In the CV of the trimetallic solution, simultaneous reduction of Pb^2+^ and Cu^2+^ started at about −1.17 V (Figure 3c; for other concentrations, see Figure S29). Similarly, Pb and Cu were deposited from a stirred trimetallic solution using −1.40 V in controlled potential coulometry at RT for about 18 h. Since these two metals do not form alloys, a simple superposition of their diffraction patterns was found in the PXRD (Figure 3d). The co‐deposited Pb/Cu has the morphology of light pink Cu visible to the naked eye on large flakes of Pb, with the flakes of Pb arranged in parallel on the substrate (Figure S30a). Electrodeposition was continued at a higher potential of −2.0 V and using a new substrate. Silvery gray Zn covered uniformly the Cu substrate (Figure 3d), with more Zn subsequently adhering to the Zn in clusters (Figure S30b). The solution's color changed from light blue to light brown and then dark brown (Figure S30c). After the AN‐based electroseparation, only 17.8 % of [Zn(bet)_2_]^2+^ and in total 8.6 % of [Pb(bet)_2_]^2+^ and [Cu_2_(bet)_4_(NTf_2_)_2_]^2+^ remained in solution (Figure S30d).

For the bimetallic solution of [Zn(bet)_2_][NTf_2_]_2_ and [Cu_2_(bet)_4_(NTf_2_)_2_][NTf_2_]_2_, simply from the green CV data of Cu‐betaine complex, the CV curve starts a gradual decrease from −1.25 V (Figure S31b) or −1.24 V (Figure S29c), where Cu deposition occurs. As described above, the electrodeposition of Cu occurs at −1.17 V (Figure S28). The small difference in voltage is due to acceptable experimental error. Then, at about −1.64 V, Zn^2+^ is reduced to Zn (blue line in Figure S31a). Thus, the bimetallic Zn‐Cu system can also effect efficiently concentrated electroseparation. Electrolysis in organic solvents (here AN) enables extremely low energy consumption and environmentally friendly deposition of common metals (here Zn, Cu, Pb). Moreover, it can also be used for efficient electroseparation of highly concentrated mixtures, which has significant implications for industrial application and technological advances in low‐temperature metal electrodeposition.^[3]^

### Characterization of Zn/^3D^ *X* (*X*=CF, Cu, Ni) anodes

Environmentally friendly Zn plating/stripping with fast reaction kinetics is well suited for anodic electrochemical reactions in batteries.[^56^, ^57^, ^58^] However, to achieve high cycling stability and long lifetime, complex challenges from Zn anodes must be solved, especially dendrite formation.^[59]^ To overcome this problem and thus obtain uniform and dense Zn deposition, various methods such as addition of inorganic, organic or polymeric additives,^[60]^ 3D architecture Zn,^[3]^ backside‐plating Zn, water‐in‐salt electrolyte, construction of artificial solid‐electrolyte interfaces, ionic liquids containing nickel triflate, or metal‐organic skeletal host have been used to control the growth of electrochemically deposited Zn.[^60^, ^61^, ^62^, ^63^, ^64^, ^65^, ^66^] However, additives usually exacerbate polarization or are expensive or even harmful to the environment, so it becomes more critical to construct Zn/^3D^ *X* (*X*=CF, Cu, or Ni) to increase the anode surface area and thus reduce the anode current density to retard the dendrite growth time.[^3^, ^59^] Consequently, the method for the preparation of 3D Zn anodes by facile electrodeposition from [Zn(bet)_2_][NTf_2_]_2_/AN solutions will appear to be essential.

**Figure 4:**
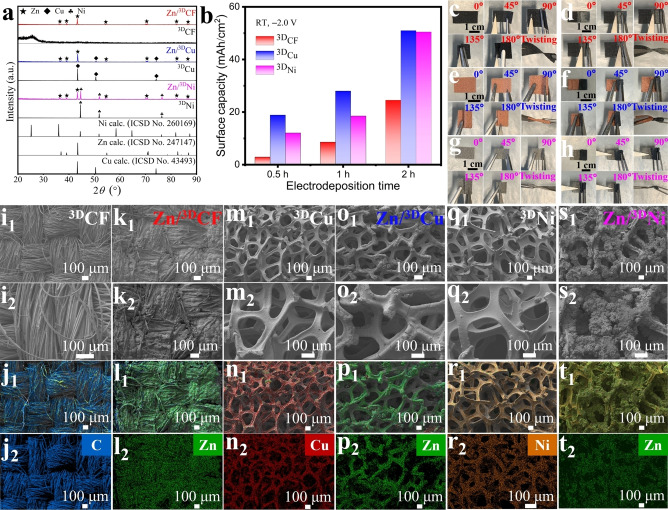
Characterization of Zn/^3D^CF (d,k_1_,k_2_,l_1_,l_2_), Zn/^3D^Cu (f,o_1_,o_2_,p_1_,p_2_), and Zn/^3D^Ni (h,s_1_,s_2_,t_1_,t_2_) anodes electrodeposited at −2.0 V and RT from 0.5 m [Zn(bet)_2_][NTf_2_]_2_/AN solutions. (a) Comparison of PXRD patterns of electrodeposited Zn layer with corresponding substrates. (b) Surface capacity versus electrodeposition time on 3D substrates. (c–h) Flexibility characterization of ^3D^CF (c), ^3D^Cu (e), and ^3D^Ni (g) substrates and the Zn‐covered 3D anodes (d,f,h) by bending and twisting experiments. (i–t) SEM images and EDX elemental mappings of the C, Cu, Ni, or Zn distribution of ^3D^CF (i_1_,i_2_,j_1_,j_2_), Zn/^3D^CF (k_1_,k_2_,l_1_,l_2_), ^3D^Cu (m_1_,m_2_,n_1_,n_2_), Zn/^3D^Cu (o_1_,o_2_,p_1_,p_2_), ^3D^Ni (q_1_,q_2_,r_1_,r_2_), and Zn/^3D^Ni (s_1_,s_2_,t_1_,t_2_).

^3D^CF appears as a black fabric (Figure 4c), crossed by carbon fibers with a diameter of approximately 11 to 15 μm (Figure 4i_1_,i_2_,j_1_,j_2_ and Figure S32), with good flexibility, which can be bent and twisted at will without permanent deformation (Figure 4c). ^3D^Cu (Figure 4m_1_,m_2_,n_1_,n_2_ and Figure S33) and ^3D^Ni (Figure 4q_1_,q_2_,r_1_,r_2_ and Figure S34) have similar 3D ductile skeletal structures with abundant interconnecting voids, resulting in a labyrinth with large surface area. Although the ^3D^Cu (Figure 4e) and ^3D^Ni (Figure 4g) substrates can still be bent at different angles and twisted further, irreversible creases will emerge. Using ^3D^CF, ^3D^Cu and ^3D^Ni as the working electrodes respectively, the corresponding Zn‐loaded 3D anodes can be prepared using the same three‐electrode system. The PXRD data (Figure 4a) show that the electrodeposition of pure crystalline Zn can be obtained on all three substrates, but their morphology is significantly different. In the case of ^3D^CF, the very dense Zn layer covers almost all of the carbon fibers and inter‐fiber gaps (Figure 4k_1_,k_2_ and Figure S35) without any impurity elements (Figure 4l_1_,l_2_ and Figure S36). Although covered with a thick Zn layer, the high flexibility of Zn/^3D^CF anode persists (Figure 4d), making it a good candidate for a flexible Zn anodes.^[67]^ In the case of ^3D^Cu, the dense Zn layer is tightly wrapped around the Cu skeleton, but the labyrinth is basically retained (Figure 4o_1_,o_2_,p_1_,p_2_ and Figures S37 and S38). In contrast, the ^3D^Ni is only partially coated by Zn, and lumpy Zn deposits have also formed in the cavities, making the skeleton barely permeable (Figure 4s_1_,s_2_,t_1_,t_2_ and Figures S39 and S40). Further Zn depositions resulted first in ^3D^Cu and ^3D^Ni substrates completely coated by Zn, and then in Zn powders on the originally deposited Zn layer (Figure 4f,h). It is well known that all metals can be electrodeposited as powder if the current density is above a critical value.[^55^, ^68^] Both Zn/^3D^Cu and Zn/^3D^Ni, although they can still be effortlessly bent and twisted, show Zn powder shedding (Figure 4f,h).

With a ^3D^Cu substrate, not only the most uniform but also a fast Zn deposition was achieved with a Zn surface capacity of about 18.9 mAh cm^−2^ after 30 min (Figure 4b). The second best surface capacity had the ^3D^Ni substrate. The Zn/^3D^ *X* (*X*=CF, Cu, Ni) anodes can obtain good performance in organic electrolyte system based zinc batteries (Figure S41). In addition to the better performance obtained in the organic electrolyte system, in parallel, we also tested the performance of the Zn/^3D^ *X* anodes in the aqueous electrolyte base ZABs. It is worth mentioning that our focus in this article will be concentrated on the rechargeable aqueous electrolyte system based ZABs discussed next.

### Test of Zn/^3D^ *X* anodes in high‐performance rechargeable ZABs

**Figure 5:**
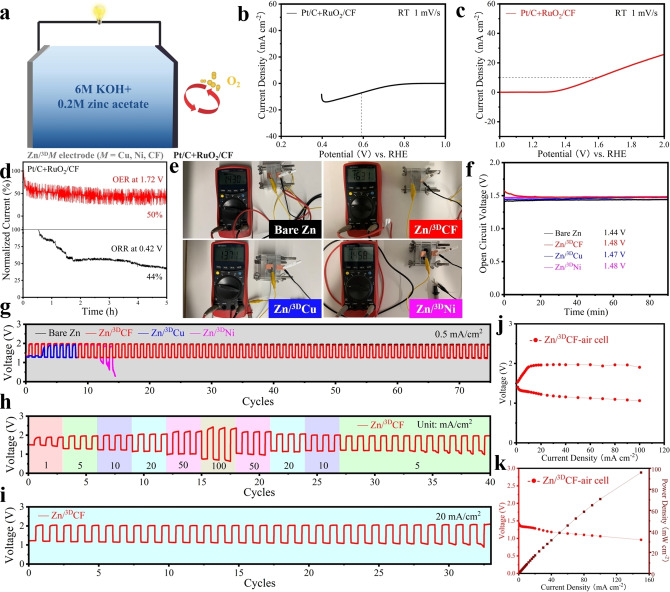
(a) Working principle and key features of ZABs. (b–d) Electrocatalytic testing of Pt/C+RuO_2_/CF: (b) ORR polarization curves. (c) OER polarization curves. (d) ORR stability evaluation at a constant potential of 0.42 V (black line) and the OER stability evaluation at a constant potential of 1.72 V (red line). (e–k) Electrochemical performance testing of aqueous rechargeable ZABs with Pt/C+RuO_2_/CF air cathode and Zn/^3D^X (X=CF, Cu and Ni) or comparative Zn foil anodes: (e) Initial open‐circuit voltage (see display). (f) Stability curve of the open‐circuit voltage within 90 min rest. (g) Galvanostatic discharge‐charge cycling curves at 0.5 mA cm^−2^, (h) rate performance of Zn/^3D^CF, and (i) the use of Zn/^3D^CF at 20 mA cm^−2^ [(g–i) charge/discharge depth was 600 s cycle^−1^]. (j) Charge and discharge polarization curves (charge/discharge depth was 3 s cycle^−1^) and (k) battery voltage and power density within Zn/^3D^CF.

The design principle for ZABs is shown in Figure 5a. The ZABs consist of a Pt/C+RuO_2_/CF electrode, acting as the air cathode in the discharge process, a Zn/^3D^ *X* electrode (*X*=Cu, Ni, CF), which is the anode during discharge, and an electrolyte composed of aqueous KOH and zinc acetate. The operating process of reversible ZABs comprises discharging and charging.^[69]^ During the discharge process, O_2_ is reduced to hydroxide ions by ORR at the air electrode, that is, at the three‐phase interface of O_2_ (gas), electrolyte (liquid), and electrocatalyst (solid). Meanwhile, the Zn anode reacts with the hydroxide ions to form Zn(OH)_4_^2−^, which is further decomposed to ZnO at supersaturated Zn(OH)_4_^2−^ concentration (Scheme 1).^[69]^ During charging, the ZABs stores energy through OER, which occurs at the air electrode/electrolyte interface, while Zn is deposited on the cathode surface through the reduction of ZnO (Scheme 1).^[69]^

To evaluate the electrocatalytic activity of the Pt/C+RuO_2_/CF bifunctional oxygen electrocatalyst, a three‐electrode system was used in 1 m KOH solution. For ORR, the halfwave potential (*E*_1/2_) of Pt/C+RuO_2_/CF was about 0.59 V (Figure 5b). The ORR durability of Pt/C+RuO_2_/CF was evaluated at a constant potential of 0.42 V. As shown by the black curve in Figure 5d, the ORR current density decreased to 44 % after 5 h. When the current density (*J*) was 10 mA cm^−2^ (Figure 5c), the potential was about 1.60 V. During the OER stability test at 1.72 V for 5 h, the OER current density dropped to 50 % (Figure 5d). Dense bubbles of O_2_ were continuously and evenly produced on the CF (Figure S42a). The overall performance of Pt/C+RuO_2_/CF was better than those of Pt/C+RuO_2_/Cu and Pt/C+RuO_2_/Ni from the experimental data (Figure S43d,e vs. Figure 5d). In addition, after the OER experiment, both Pt/C+RuO_2_/Cu and Pt/C+RuO_2_/Ni showed shedding of Pt/C+RuO_2_ active substances, owing to the continuous generation of O_2_. The bifunctional oxygen activity of an electrocatalyst is usually measured as the potential difference Δ*E* between *E*_*J*=10_ and *E*_1/2_ (Δ*E*=*E*_*J*=10_ – *E*_1/2_, where *E*_*J*=10_ is the OER potential at 10 mA cm^−2^ and *E*_1/2_ is the ORR halfwave potential).^[45]^ Accordingly, the Δ*E* of Pt/C+RuO_2_/CF is about 1.01 V (Figure S42b).

The homemade ZABs (Figure 5e) were assembled using a Pt/C+RuO_2_/CF air cathode and 3D Zn/^3D^ *X* anodes with different substrates ^3D^ *X* (Figure 4d,f,h) or for reference bare Zn foil. Remarkably, compared to Zn foil and other ^3D^ *X* substrates, the ZABs with Zn/^3D^CF anodes reached a very large open‐circuit voltage of 1.631 V (Figure 5e). Also, when compared to reported ZABs with Co‐based electrocatalysts (1.576 V for NP‐Co_3_O_4_/CC),^[45]^ the initial open‐circuit voltage is very competitive. This illustrates with experimental evidence that optimizing the anode is essential for increasing the performance of ZABs.

ZAB assembly is accomplished, after 90 min rest, the ZAB with the Zn/^3D^CF anode reached a stable voltage of 1.48 V, similar to the other tested ZABs (Figure 5f). More convincing, EIS results show that the initial Zn/^3D^CF anode based ZAB has an impedance of 1.26 Ω that is only about one tenth of the 10.97 Ω impedance of the Zn foil. Although not as good as Zn/^3D^CF anode, ^3D^Cu and ^3D^Ni substrates also have the effect of improving the kinetics of ZABs (Figure S44). The impedance of the ZAB with Zn/^3D^CF anode stabilized at 1.18 Ω after 90 min rest (Figure S44b). We attribute the slight reduction of the impedance to the complete wetting of the electrolyte. These excellent kinetics will help to improve the cycling performance of the ZABs significantly. In contrast, the slight increase in impedance for Zn/^3D^Cu and loose Zn/^3D^Ni anodes base ZABs is due to the rapid Zn shedding problem in electrolyte (Figure S44c,d).

The ZAB with Zn/^3D^CF anode showed good cycling stability with a very smooth potential even after 75 cycles (Figure 5g) and a constantly dense morphology of the anode (Figure 4k_1_,k_2_). In contrast, the Zn/^3D^Cu and Zn/^3D^Ni anode failed during seen by the large potential difference greater than 3.5 V. The failure of the Zn/^3D^Cu and Zn/^3D^Ni anodes in the alkaline electrolyte was accompanied by shedding of Zn powder.

More notably, the ZAB with Zn/^3D^CF anode also revealed good rate performance (Figure 5h). Full reversibility was observed when ramping the current density from 1 mA cm^−2^ to 100 mA cm^−2^ and followed by continuous cycling at 5 mA cm^−2^. At constant current densities of 5 mA cm^−2^ (Figure S45) or even 20 mA cm^−2^ (Figure 5i), the ZAB with Zn/^3D^CF anode can be cycled very stably.

As shown in Figure 5j, the overall charge‐discharge voltage gap of Pt/C+RuO_2_/CF is 0.84 V at a current density of 100 mA cm^−2^. The power density and discharge polarization curves of individual cells (Figure 5k) show that Pt/C+RuO_2_/CF has a peak power density of about 96 mW cm^−2^.

**Figure 6:**
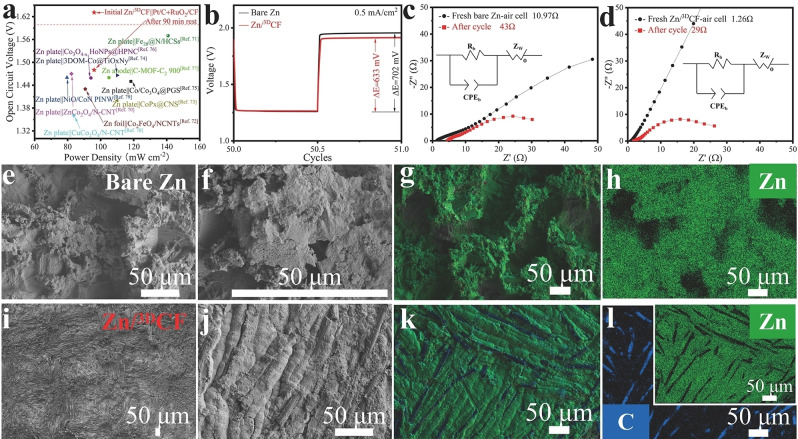
(a) Open‐circuit voltage and power density of the Zn/^3D^CF anode‐based battery compared to other reported ZABs. (b) Galvanostatic discharge‐charge cycling curves of the 50^th^ cycle at 0.5 mA cm^−2^ (charge/discharge depth: 600 s cycle^−1^; enlargement of Figure 5g). (c,d) EIS before and after 75 cycles for bare Zn (c) and the Zn/^3D^CF anode (d); inset: the equivalent circuit for the simulation. (e–l) SEM images and EDX elemental mappings of the C or Zn distribution of cycled bare Zn (e–h) and Zn/^3D^CF anode (i–l).

The galvanostatic discharge‐charge cycling curves of the 50^th^ cycle at 0.5 mA cm^−2^ demonstrates that the Zn/^3D^CF anode has a lower 633 mV overpotential compared to the 702 mV of bare Zn (Figure 6b). After 75 cycles, the impedance of the ZABs with bare Zn or Zn/^3D^CF anodes had increased due to the loss of Zn and the production of by‐products, but the impedance of the ZAB with bare Zn anode (Figure 6c) was still about 1.5 times higher than that with a Zn/^3D^CF anode (Figure 6d). After cycling, the morphology of the bare Zn anode showed many fractures, pits, pores, corrosion, and dendrite growth in some areas, making the surface very rough (Figure 6e–h, Figures S46 and S47). In contrast, the surface of the Zn/^3D^CF anode remained relatively flat (Figure 6i–l, Figures S48 and S49). Not only in terms of open‐circuit voltage and power density, our ZAB with Zn/^3D^CF anode is very competitive with previously reported rechargeable ZABs (Figure 6a and Table S1).[^70^, ^71^, ^72^, ^73^, ^74^, ^75^, ^76^, ^77^, ^78^, ^79^] We attribute the good performance to our binder‐free Zn/^3D^CF anode that shows excellent kinetics and inhibits the growth of dendrites.

## Conclusion

Electrodeposition in organic solvents allows efficient separation of Zn from Pb and Cu, which are typical co‐metals in natural resources and/or wastes, even from concentrated solutions of their betaine complexes, which can be easily obtained from the metal oxides. The highest current efficiency (86 %) among the solvents tested was obtained for acetonitrile. The morphology of the electrodeposited metal can be influenced by the choice of solvent. This kind of electrodeposition can become an ecologically and economically attractive alternative compared to conventional metallurgical processes. Regardless of future large‐scale applications, metal electrodeposition from organic electrolytes can be used immediately, as we have demonstrated in the preparation of a Zn/^3D^CF anode with superior kinetics (cell impedance) and inhibition of dendrite formation. In a rechargeable zinc‐air battery it proved competitive cycling stability and rate performance.

## Experimental Section

### Chemicals and materials

[Hbet]Cl (99 %) was purchased from Alfa Aesar. Lead oxide (PbO, 99 %) was supplied by Sigma Aldrich. Copper oxide (CuO, 99.995 %) were obtained from Alfa Aesar. AN (<30 ppm H_2_O) was obtained from VWR chemicals and sulfolane (99 %) from Merck. PC (anhydrous, 99.7 %) was supplied by Sigma Aldrich. Acetone (ACS reagent, ≥99.5 %) and DMF (for HPLC, ≥99.9 %) were bought from Sigma Aldrich. Methanol (gradient grade) was obtained from VWR chemicals. Ethanol (absolute, analytical reagent grade) was purchased from Fisher Chemical. Ruthenium(IV) oxide (RuO_2_, 99.9 % trace metals basis) was supplied by Sigma Aldrich. Pt/C (80 %) was purchased from FuelCellStore. Perfluorinated resin solution containing Nafion (Nafion, 5 wt.% in lower aliphatic alcohols and water, contains 15 to 20 % water) was purchased from Sigma Aldrich. Potassium hydroxide (KOH, analytical reagent grade) was purchased from Fisher Chemical. ZnO (p. a.) was obtained from Grillo. Lithium hexafluorophosphate (LiPF_6_, battery grade, ≥99.99 %) and ethylmethylcarbonate (EMC, 99.9 %) were purchased from Sigma Aldrich. Li[NTf_2_] (80 % solution in water) was purchased from Iolitec. Zn[NTf_2_]_2_ (99.5 %) was a product of Solvionic company. Zn foil (ca. 250 μm) was bought from Alfa Aesar. Cu foil (12 μm thickness), ^3D^Cu foam, 3D nickel foam (^3D^Ni), ^3D^CF and zinc acetate dehydrate (Zn(OAc)_2_) were laboratory grade. All chemicals were used as received without further purification or treatment.

### Preparation of [Hbet][NTf_2_]

The IL [Hbet][NTf_2_] was synthesized by using a reported ion‐exchange process.^[3]^ At first, 0.1 mol of [Hbet]Cl and a solution equivalent to 0.1 mol of Li[NTf_2_] were dissolved in 250 ml of deionized water and stirred for 1 h. Subsequently, [Hbet][NTf_2_] was separated and washed five times with ice water until detection with silver nitrate solution indicated the absence of chloride ions. Then, [Hbet][NTf_2_] was dried overnight using a Schlenk line at 130 °C.

### Synthesis of [*M*(bet)_2_][NTf_2_]_2_ (*M*=Zn, Pb), [Cu_2_(bet)_4_(NTf_2_)_2_][NTf_2_]_2_ and their mixtures

The synthesis of [*M*(bet)_2_][NTf_2_]_2_ with *M*=Zn or Pb was performed by stirring the metal oxide and [Hbet][NTf_2_] in the molar ratio of *n*_MO_/*n*_IL_=1 : 2 (*M*O=metal oxide) and heating to 175 °C in an flask open to the air for 12 h, and after that, drying at 175 °C for 12 h using a Schlenk line. For the CuO system, a mass ratio of *m*_CuO_/*m*_IL_=124.8 mg:2.5 g (*n*_CuO_/*n*_IL_=1 : 4) and additional about 1.2 mg [Hbet]Cl were also prepared according to the method described above.^[7]^ Similarly, according to the above‐mentioned methods, solutions for the (Zn, Pb) and (Zn, Pb/Cu) electroseparation system were prepared in mass ratios of *m*_ZnO_/*m*_PbO_/*m*_IL_=204 mg : 280 mg:2 g (*n*_ZnO_/*n*_PbO_/*n*_IL_≈2 : 1 : 4) and *m*_ZnO_/*m*_PbO_/*m*_CuO_/*m*_[Hbet]Cl_/*m*_IL_=204 mg : 280 mg : 13.1 mg : 1.0 mg : 3 g (*n*_ZnO_/*n*_PbO_/*n*_CuO_/*n*_IL_≈15 : 7.69 : 1 : 46). The PXRD pattern of ZnO used in this experiment is shown in Figure S50.

### Synthesis of electrolytes for electrodeposition

All electrolytes were prepared in atmospheric environment at RT. [Zn(bet)_2_][NTf_2_]_2_ was completely dissolved in AN, acetone, DMF, methanol, PC and sulfolane to give a transparent solution of 0.5 m by stirring for 1 h. Solutions of [Pb(bet)_2_][NTf_2_]_2_ and [Cu_2_(bet)_4_(NTf_2_)_2_][NTf_2_]_2_ in AN at 0.5 m can also be obtained according to the method described above. Additional, highly concentrated solutions of Zn, Pb and Zn, Pb/Cu electroseparation are prepared by adding 5 g AN to the above prepared mixtures of Zn, Pb or Zn, Pb, and Cu complex compounds, respectively. Ultimately, solutions of *m* ${}_{\left[\mathrm{Zn}\left(\mathrm{bet}\right){}_{2}\right]\left[\mathrm{NTf}{}_{2}\right]{}_{2}}$ /*m* ${}_{\left[\mathrm{Pb}\left(\mathrm{bet}\right){}_{2}\right]\left[\mathrm{NTf}{}_{2}\right]{}_{2}}$ /*m*_AN_≈2.156 g : 1.2567 g : 5 g (*n* ${}_{\mathrm{Zn}\left(\mathrm{bet}\right){}_{2}]\left[\mathrm{NTf}{}_{2}\right]{}_{2}}$ /*n* ${}_{\left[\mathrm{Pb}\left(\mathrm{bet}\right){}_{2}\right]\left[\mathrm{NTf}{}_{2}\right]{}_{2}}$ /*n*_AN_≈2:1 : 97) and *m* ${}_{\left[\mathrm{Zn}\left(\mathrm{bet}\right){}_{2}\right]\left[\mathrm{NTf}{}_{2}\right]{}_{2}}$ /*m* ${}_{\left[\mathrm{Pb}\left(\mathrm{bet}\right){}_{2}\right]\left[\mathrm{NTf}{}_{2}\right]{}_{2}}$ /*m* ${}_{\left[\mathrm{Cu}{}_{2}\left(\mathrm{bet}\right){}_{4}\left(\mathrm{NTf}{}_{2}\right){}_{2}\right]\left[\mathrm{NTf}{}_{2}\right]{}_{2}}$ /*m*_AN_≈2.156 g : 1.2567 g : 0.1415 g : 5 g (*n* ${}_{\mathrm{Zn}\left(\mathrm{bet}\right){}_{2}]\left[\mathrm{NTf}{}_{2}\right]{}_{2}}$ /*n* ${}_{\left[\mathrm{Pb}\left(\mathrm{bet}\right){}_{2}\right]\left[\mathrm{NTf}{}_{2}\right]{}_{2}}$ /*n* ${}_{\left[\mathrm{Cu}{}_{2}\left(\mathrm{bet}\right){}_{4}\left(\mathrm{NTf}{}_{2}\right){}_{2}\right]\left[\mathrm{NTf}{}_{2}\right]{}_{2}}$ /*n*_AN_≈2 : 1:0.06 : 97) were obtained. Traces of unreacted CuO may still be present in the trimetallic mixture after dissolution in AN. In this case, the solution was centrifuged at 4000 rpm for 4 min and the supernatant blue liquid was taken for subsequent electroseparation experiments.

### Preparation of hybrid electrolytes for symmetric batteries, Pt/C+RuO_2_/*X* (*X*=CF, Cu, Ni) air cathode and Zn/^3D^ *X* anode

The hybrid electrolytes were prepared in an argon‐filled glove box with water and oxygen levels below 0.1 ppm. LiPF_6_ and Zn[NTf_2_]_2_ were completely dissolved in EMC and stirred in the glove box for 8 h to obtain solutions with concentrations of 2 m and 0.5 m, respectively. The Pt/C and RuO_2_ ink dispersion was prepared by mixing 2.5 mg Pt/C, 2.5 mg RuO_2_, 50 μL Nafion solution and 1 ml of ethanol. The Pt/C and RuO_2_ ink was dropped onto the cleaned CF with a mass loading of about 1.03 mg cm^−2^, after evaporation of all ethanol overnight. Pt/C+RuO_2_/*X* (*X*=Cu, Ni) electrodes were also prepared by using same method (Figure S43b,c). The mass loadings of Pt/C+RuO_2_ on Cu and Ni are about 1.35 mg cm^−2^ and 1.5 mg cm^−2^, respectively. For the Zn/^3D^ *X* anodes (*X*=CF, Cu and Ni), Zn was electrodeposited on the three different substrates using a VMP‐3 model of Biologic SAS controlled by EC‐LAB electrochemistry software (Bio‐Logic Science Instruments) with a three‐electrode system. The electrolyte consisted of [Zn(bet)_2_][NTf_2_]_2_ and AN in the molar ratio of 0.5 m.

### Electrochemical measurements

All electrochemical experiments were performed on a VMP‐3 model of Biologic SAS controlled by EC‐LAB electrochemistry software, using a three‐electrode system at RT in the atmosphere. All electrodes were cleaned with ethanol and dried prior to measurements. The cyclic voltammograms (CV) were recorded using a three‐electrode setup (Wuhan Corrtest Instruments Corp. Ltd.). It consisted of a glassy carbon rod (GC, 3 mm diameter), a cylindrical platinum wire (Pt, 99.95 %, 0.5 mm diameter) and a Pt plate (99.95 %, 10×10×0.1 mm), which were used as the working electrode, counter electrode and reference electrode, respectively. The electrodeposition of the individual metals Zn, Pb, and Cu as well as the electroseparation of the bimetallic mixture of Zn and Pb and the trimetallic mixture were carried out by the potentiostatic method in different solvents. For the electrodeposition, copper foil (15 mm×10 mm×12 μm, for battery negative collector, only cleaned with ethanol), cylindrical Pt wire (as above) and Pt plate (as above) were used as working, counting and reference electrodes, correspondingly. For experiments with different substrates, only ^3D^CF, ^3D^Cu and ^3D^Ni were used as working electrodes, while the rest of the setup remained the same. After electrodeposition, the samples were washed first with AN and then with acetone, dried at RT and stored in an argon‐filled glove box before further characterization and use. Zn/^3D^ *X* (*X*=CF, Cu and Ni) and Zn electrodes were stamped and pressed into 12 mm diameter discs and assembled into CR2032 coin‐type cells (Manual Coin Cell Crimper AOT‐HCM‐20) in a glove box with Celgard 2400 separator and the hybrid LiPF_6_ and Zn[NTf_2_]_2_ electrolytes described above to evaluate the effective deposition of Zn on the 3D substrate. The cell was then charged galvanostatically at 0.5 mA cm^−2^ using NEWARE BTS4000‐5V 10 mA Battery Testing System (XIAMEN AOT ELECTRONICS TECHNOLOGY CO., LTD). The cell potential diverged significantly when Zn was completely stripped from the 3D substrate, as reported earlier.^[3]^ The catalytic activity of the Pt/C+RuO_2_/*X* (*X*=CF, Cu, Ni) air cathode was evaluated using a Pt wire as counter electrode and mercury/mercury oxide (Hg/HgO) electrode as reference electrode in 4 h oxygen‐purified 1 m KOH aqueous solution (pH=14) with a standard three‐electrode setup. The ORR and OER polarization curves were obtained at scanning rate of 1 mV s^−1^. The potential of Hg/HgO was converted to the reversible hydrogen electrode (RHE): *E*_RHE_=*E*_Hg/HgO_+0.059 pH+0.098. Aqueous ZABs was tested on a home‐made electrochemical device. The Pt/C+RuO_2_/CF was used directly as an air cathode. Zn/^3D^ *X* (*X*=CF, Cu and Ni, Zn loading about 200 to 250 mg) or comparative Zn foil were pressed as the anode, 6.0 m KOH and 0.2 m Zn(OAc)_2_ as the electrolytes to ensure a reversible electrochemical reaction of Zn on the anode. The electrochemical impedance spectrum (EIS) of the ZABs before and after long cycle were determined in the frequency range of 100 mHz to 1 MHz at a perturbation voltage of 10 mV.

### Powder X‐ray diffraction (PXRD)

PXRD was carried out on an Empyrean diffractometer (PAN‐analytical) at 296(1) K equipped with a curved Ge(111) monochromator in Bragg‐Brentano geometry using Cu_Kα1_ radiation (*λ*=154.0598 pm).

### Nuclear magnetic resonance spectroscopy (NMR)

The NMR spectra were recorded with a Bruker Avance Neo 300 MHz spectrometer with a 5 mm high‐resolution probe. The sample [Zn(bet)_2_][NTf_2_]_2_ was dissolved in deuterated AN. To ensure field‐frequency lock a capillary filled with [D_6_]DMSO was added to this sample. ^1^H and ^13^C NMR spectra were measured relative to tetramethylsilane. For ^13^C NMR a transmitter frequency of 75.4752953 MHz to record 512 scans with a 5 s relaxation delay.

### Scanning electron microscopy (SEM) and energy‐dispersive X‐ray (EDX) analysis

The samples were stuck to the carbon adhesive (laboratory grade). The carbon adhesive was glued on a sample holder. SEM images were recorded on a field emission scanning electron microscope (FESEM, Zeiss Gemini 500, *U*_e_=1 kV). The compositions of samples were tested by semi‐quantitative EDX analysis using a Zeiss Gemini 500 instrument equipped with an Oxford EDX detector.

### Infrared (IR) measurements and UV/Vis absorption spectrum

About 0.3 mL of these samples were dripped onto a sample table for IR measurements. Vibrational spectra were measured with a Bruker Vertex 70 FTIR spectrometer with attenuated total reflection (ATR) accessory in a radiation range from 500 to 4000 cm^−1^. Data analysis was performed with the program OPUS. Approximately 3.5–4 mL of the different liquid samples were added to the high precision cell for UV/Vis testing. UV/Vis absorption spectra were measured using a UV/Vis Spectrophotometer UV‐1650PC (SHIMADZU CORPORATION) in the wavelength range from 200 to 800 nm.

## Conflict of interest

The authors declare no conflict of interest.

1

## Supporting information

As a service to our authors and readers, this journal provides supporting information supplied by the authors. Such materials are peer reviewed and may be re‐organized for online delivery, but are not copy‐edited or typeset. Technical support issues arising from supporting information (other than missing files) should be addressed to the authors.

Supporting Information

## Data Availability

The data that support the findings of this study are available in the supplementary material of this article.
