# Supplementary material for: A Kinetically Superior Rechargeable Zinc‐Air Battery Derived from Efficient Electroseparation of Zinc, Lead, and Copper in Concentrated Solutions
Source: ChemSusChem. 2022 Apr 20;15(10):e202200039. doi: 10.1002/cssc.202200039 (PMC9325370; doi:10.1002/cssc.202200039)
Supplement: Supplementary file 1 — Supporting Information [file CSSC-15-0-s001.pdf]

ChemSusChem

Supporting Information

A Kinetically Superior Rechargeable Zinc-Air Battery Derived from Efficient Electroseparation of Zinc, Lead, and Copper in Concentrated Solutions

Peng Chen, Xia Wang, Dongqi Li, Tobias Pietsch, and Michael Ruck*© 2022 The Authors. ChemSusChem published by Wiley-VCH GmbH. This is an open access article under the terms of the Creative Commons Attribution License, which permits use, distribution and reproduction in any medium, provided the original work is properly cited.

Table of Contents

Figure S1 3
Figure S2 3
Figure S3 4
Figure S4 4
Figure S5 5
Figure S6 5
Figure S7 6
Figure S8 6
Figure S9 7
Figure S10 7
Figure S11 8
Figure S12 8
Figure S13 9
Figure S14 9
Figure S15 9
Figure S16 10
Figure S17 10
Figure S18 11
Figure S19 11
Figure S20 12
Figure S21 12
Figure S22 12
Figure S23 13

Figure S24 13
Figure S25 14
Figure S26 14
Figure S27 15
Figure S28 15
Figure S29 16
Figure S30 16
Figure S31 17
Figure S32 17
Figure S33 18
Figure S34 18
Figure S35 19
Figure S36 19
Figure S37 20
Figure S38 20
Figure S39 21
Figure S40 21
Figure S41 22
Figure S42 22
Figure S43 23
Figure S44 23
Figure S45 24
Figure S46 24
Figure S47 24
Figure S48 25
Figure S49 25
Figure S50 26
Table S1 26
References 27

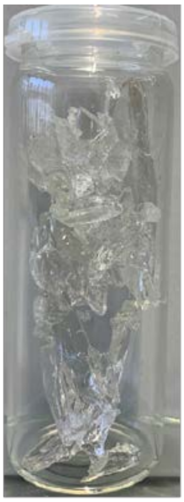

Figure S1. Photograph of $[Zn(bet)_2][NTf_2]_2$ at room temperature.

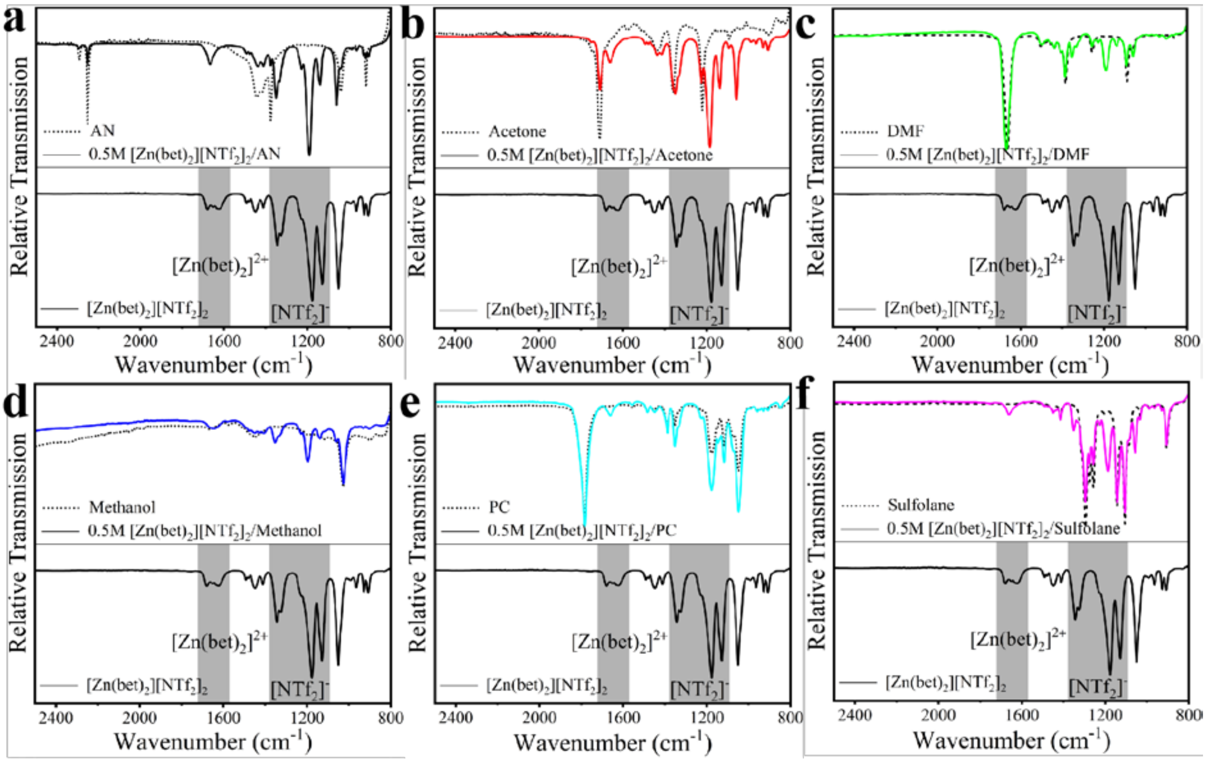

Figure S2. FT-IR spectra of six different solutions compare to pure $[Zn(bet)_2][NTf_2]_2$ in the range of 800 $cm^{-1} \leq \bar{\nu} \leq 2500$ cm^{-1} (enlarged images of Figure 1d).

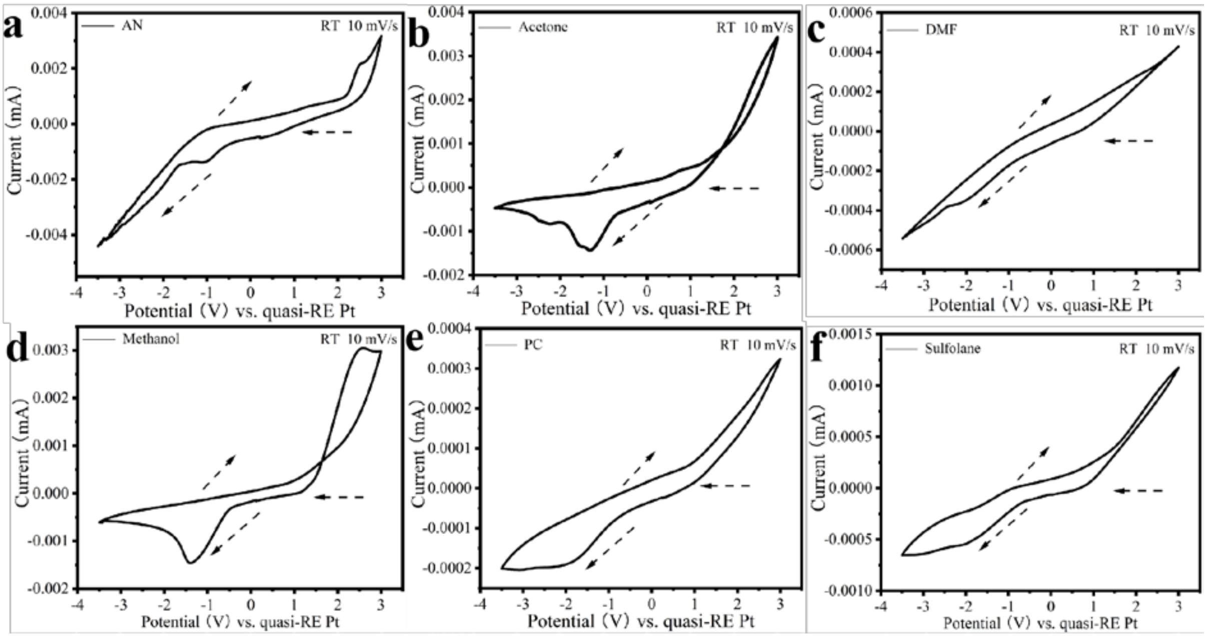

Figure S3. CVs of pure organic solvents at a scan rate of 10 mV s^{-1} and RT: **(a)** acetonitrile (AN), **(b)** acetone, **(c)** dimethylformamide (DMF), **(d)** methanol, **(e)** propylene carbonate (PC), **(f)** sulfolane.

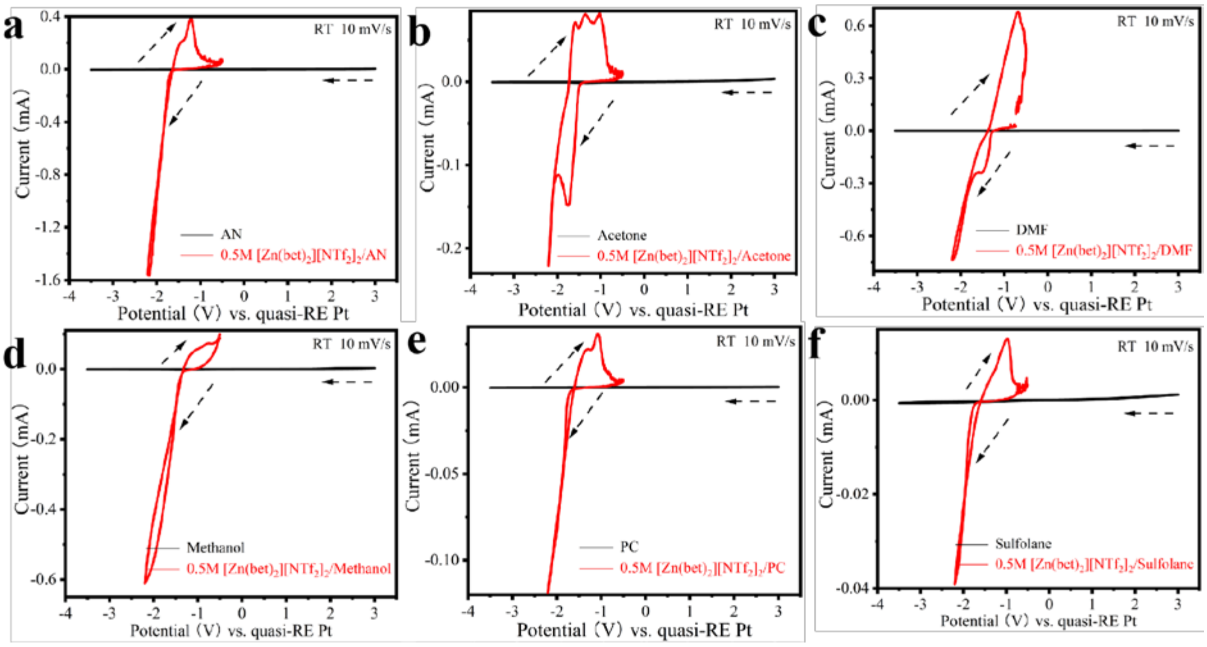

Figure S4. The CV comparison between pure organic solvents and six different 0.5 M electrolytes at a scan rate of 10 mV s^{-1} and RT (compared to Figure S3 is a different y-axis current range). **(a)** $[Zn(bet)_2][NTf_2]_2$/AN solutions. **(b)** $[Zn(bet)_2][NTf_2]_2$/acetone. **(c)** $[Zn(bet)_2][NTf_2]_2$/DMF. **(d)** $[Zn(bet)_2][NTf_2]_2$/methanol. **(e)** $[Zn(bet)_2][NTf_2]_2$/PC. **(f)** $[Zn(bet)_2][NTf_2]_2$/sulfolane.

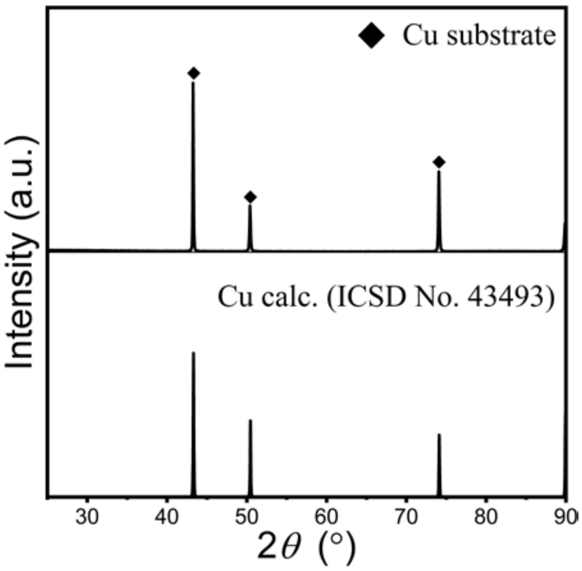

Figure S5. Comparison of the PXRD pattern of the Cu foil substrate used in this work with the reference.

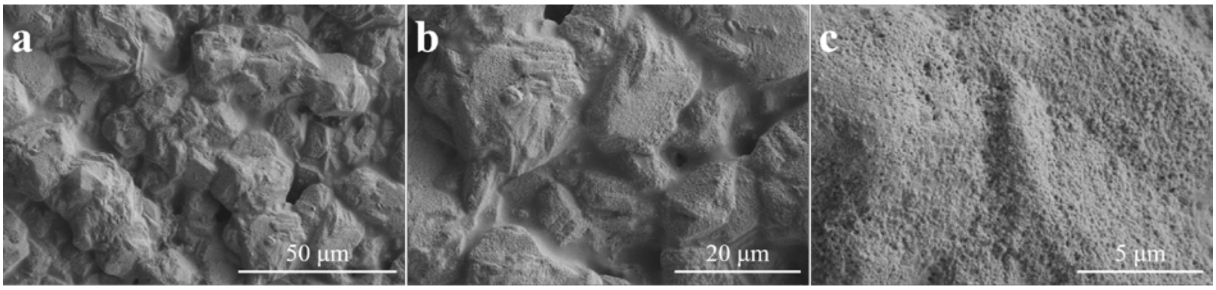

Figure S6. (a-c) SEM images (increasing magnification) of electrodeposited Zn on Cu foil substrate at –2.0 V and RT after about 5 h from 0.5 M $[Zn(bet)_2][NTf_2]_2$/DMF solutions.

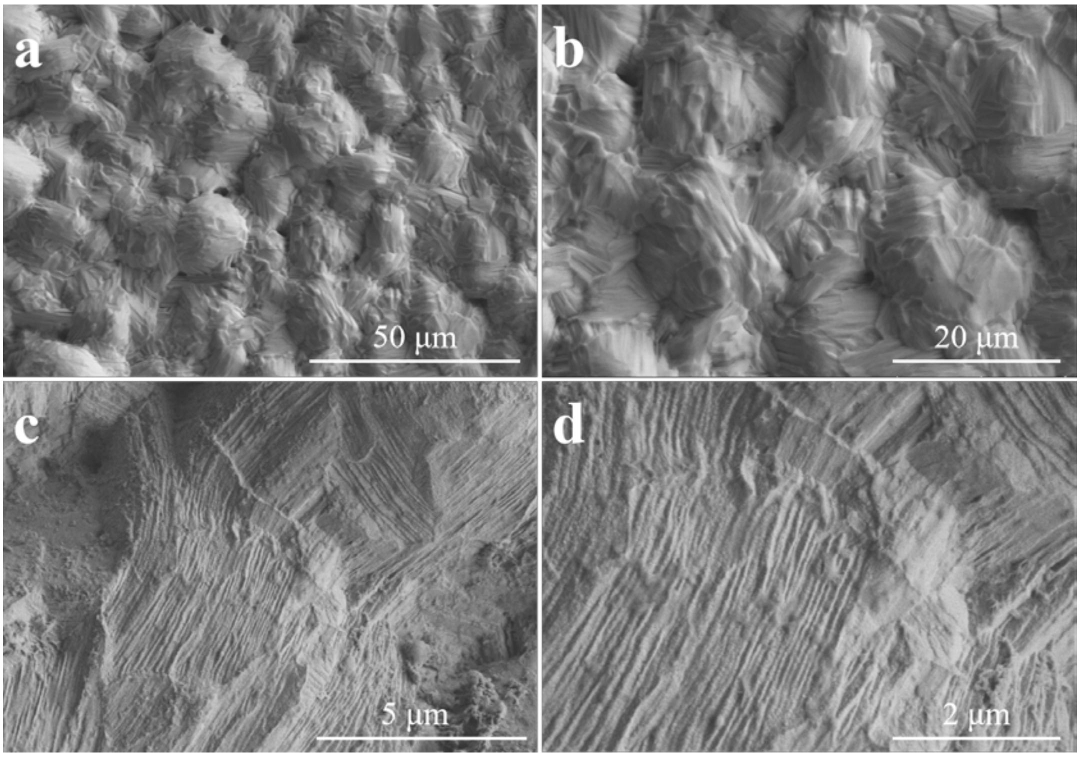

Figure S7. (a-d) SEM images (increasing magnification) of electrodeposited Zn on Cu foil substrate at –2.0 V and RT after about 5 h from 0.5 M $[Zn(bet)_2][NTf_2]_2$/PC solutions.

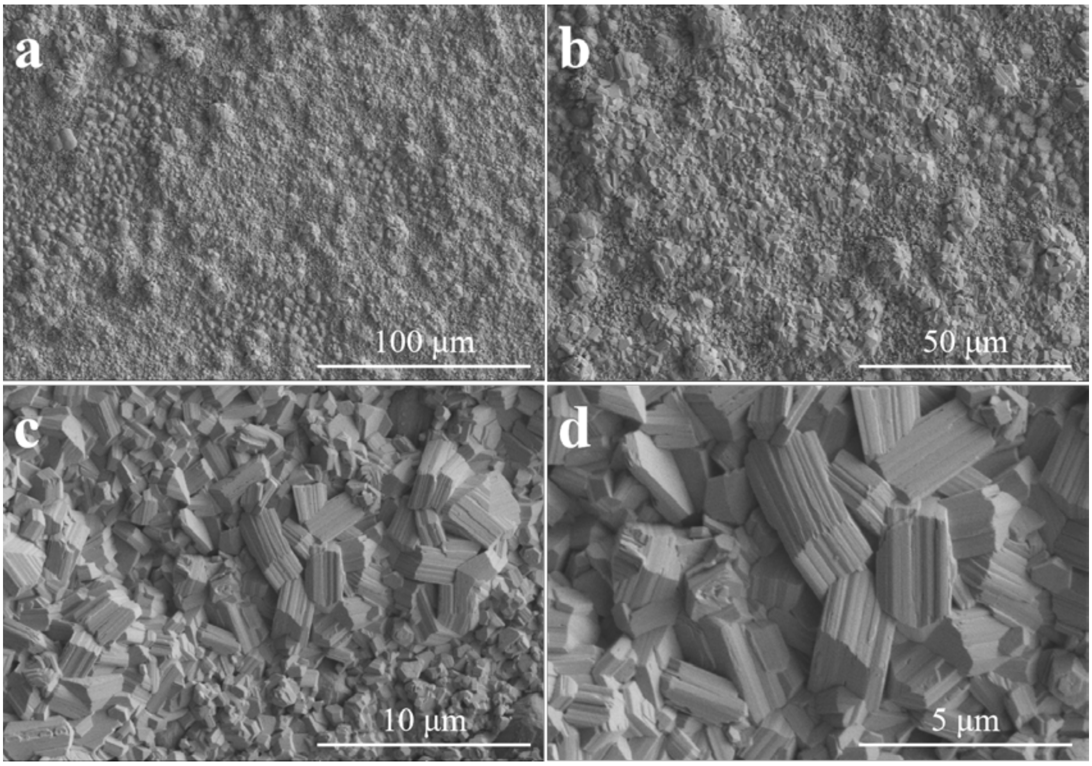

Figure S8. (a-d) SEM images (increasing magnification) of electrodeposited Zn on Cu foil substrate at –2.0 V and RT after about 5 h from 0.5 M $[Zn(bet)_2][NTf_2]_2$/sulfolane solutions.

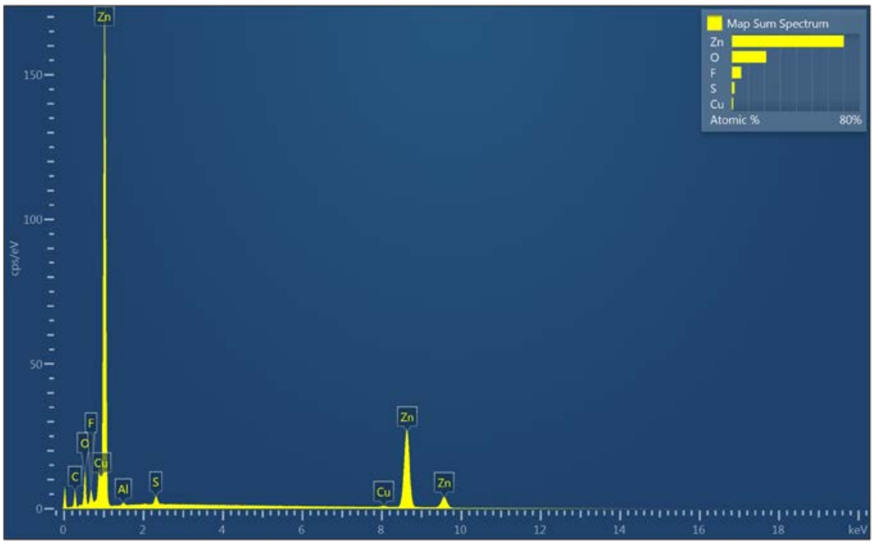

Figure S9. EDX analysis of electrodeposited Zn on Cu foil substrate at –2.0 V and RT after about 5 h from 0.5 M $[Zn(bet)_2][NTf_2]_2$/DMF solutions.

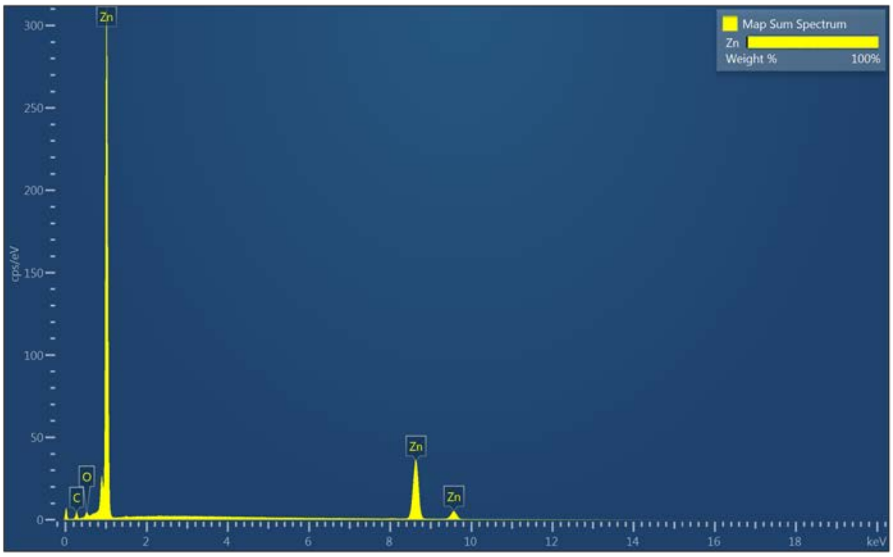

Figure S10. EDX analysis of electrodeposited Zn on Cu foil substrate at –2.0 V and RT after about 5 h from 0.5 M $[Zn(bet)_2][NTf_2]_2$/PC solutions.

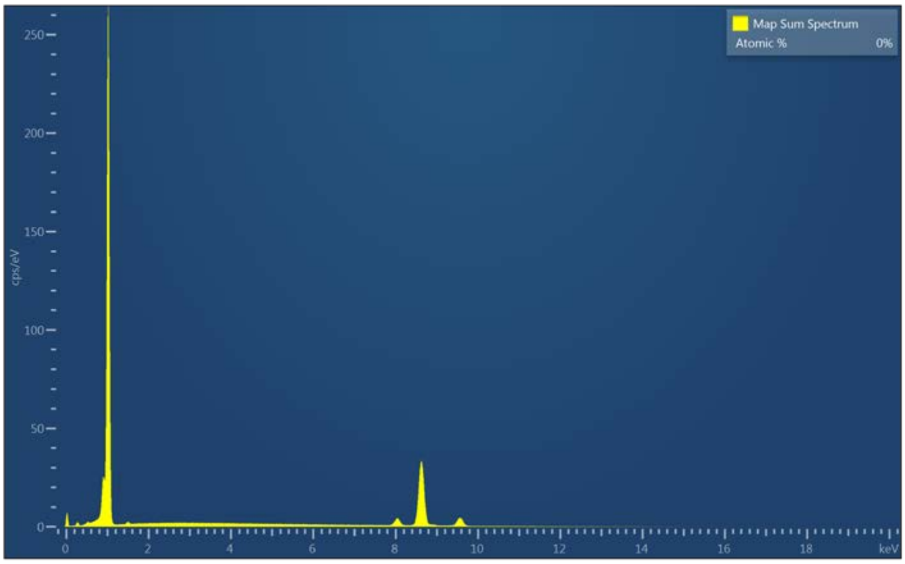

Figure S11. EDX analysiss of electrodeposited Zn on Cu foil substrate at –2.0 V and RT after about 5 h from 0.5 M [Zn(bet)$_2$][NTf$_2$]$_2$/sulfolane solutions.

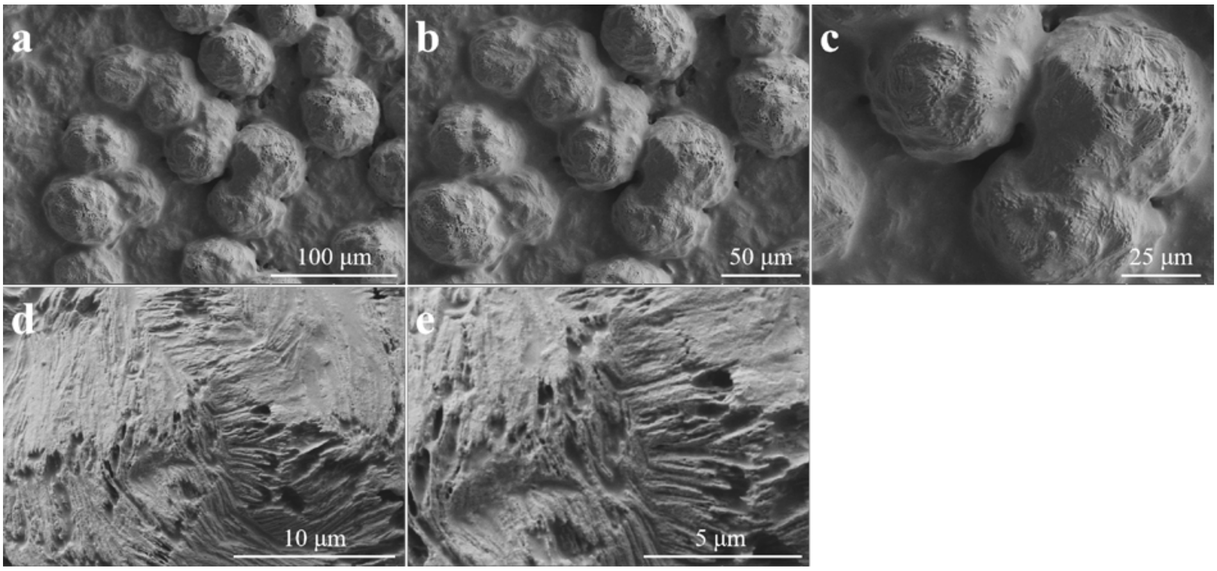

Figure S12. (a-e) SEM images (increasing magnification) of electrodeposited Zn on Cu foil substrate at –2.0 V and RT after about 5 h from 0.5 M [Zn(bet)$_2$][NTf$_2$]$_2$/acetone solutions.

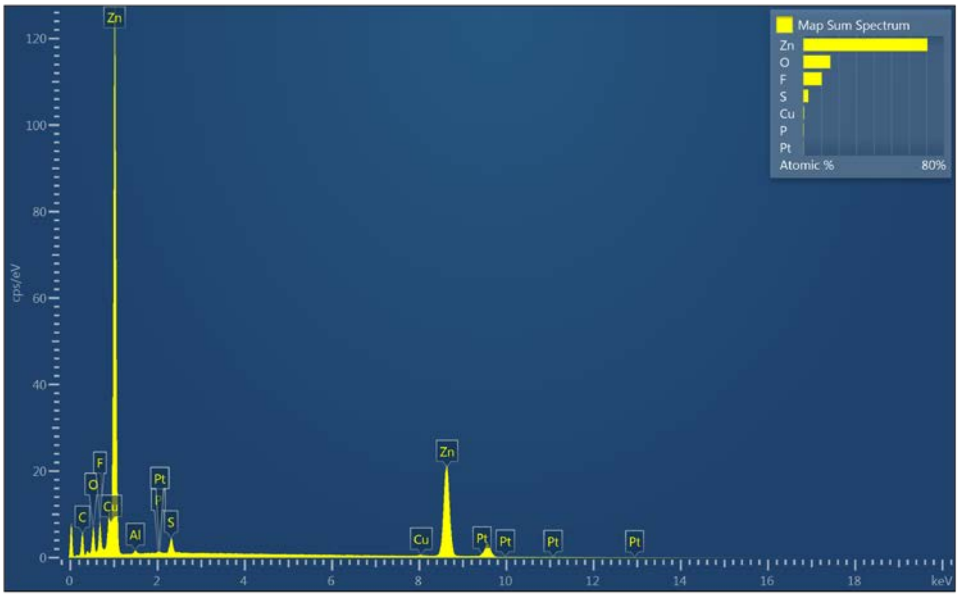

Figure S13. EDX analysis of electrodeposited Zn on Cu foil substrate at –2.0 V and RT after about 5 h from 0.5 M $[Zn(bet)_2][NTf_2]_2$/acetone solutions.

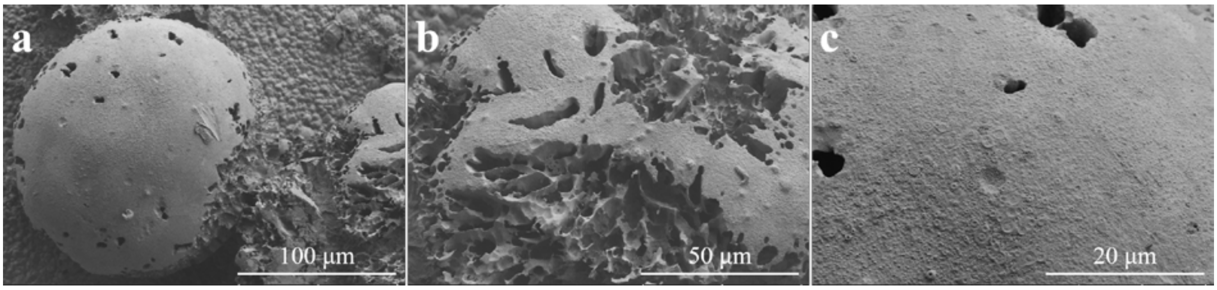

Figure S14. (a-c) SEM images (increasing magnification) of electrodeposited Zn on Cu foil substrate at –2.0 V and RT after about 5 h from 0.5 M $[Zn(bet)_2][NTf_2]_2$/methanol solutions.

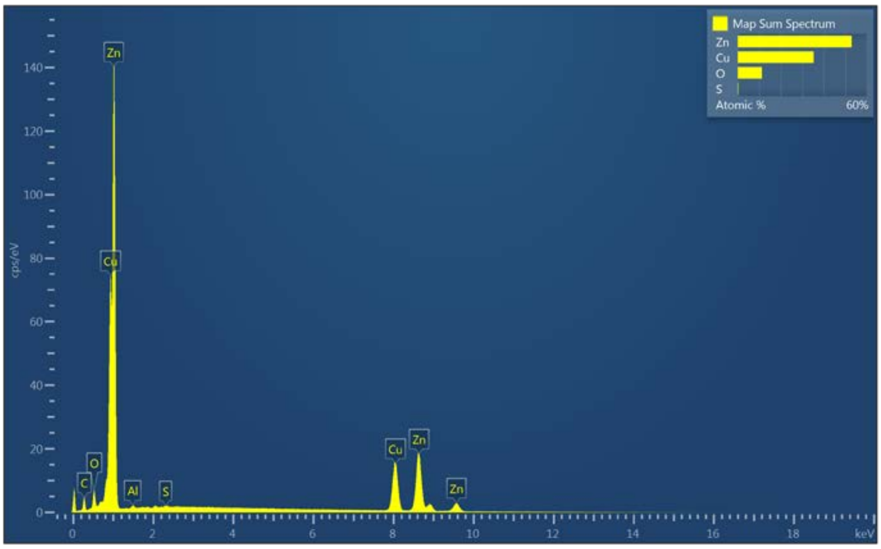

Figure S15. EDX signals of electrodeposited Zn on Cu foil substrate at –2.0 V and RT after about 5 h from 0.5 M $[Zn(bet)_2][NTf_2]_2$/methanol solutions.

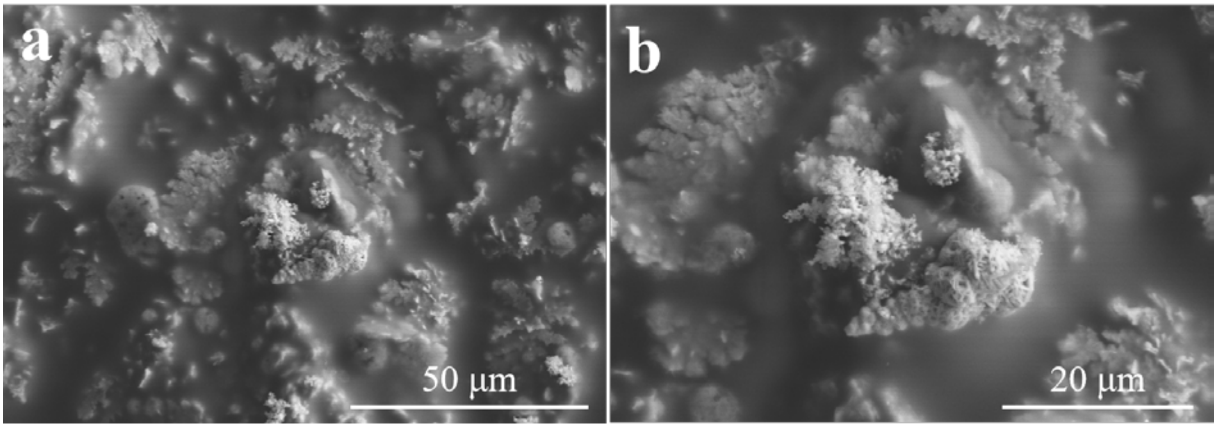

Figure S16. (a-b) SEM images (increasing magnification) of electrodeposited Zn on Cu foil substrate at –2.0 V and RT after about 5 h from 0.5 M $[Zn(bet)_2][NTf_2]_2$/AN solutions.

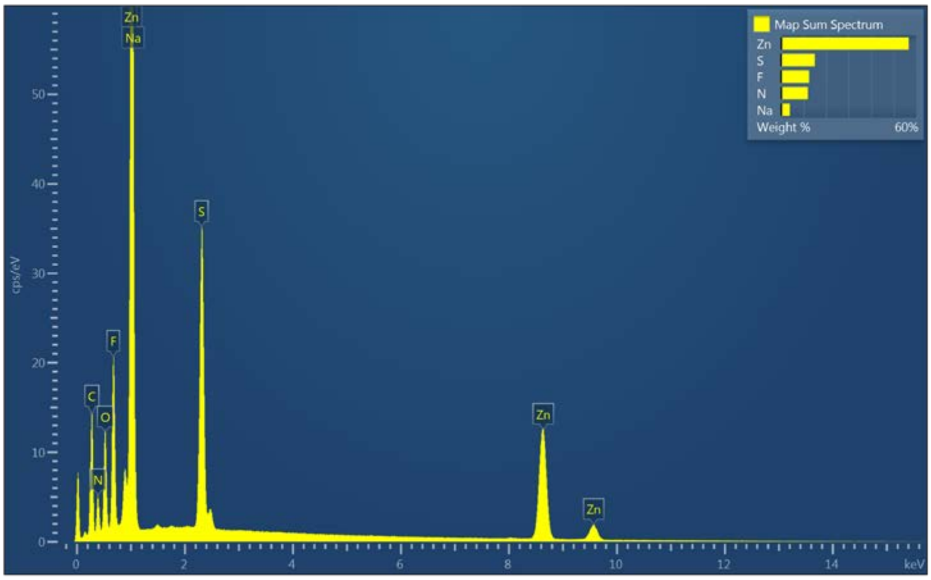

Figure S17. EDX analysis of electrodeposited Zn on Cu foil substrate at –2.0 V and RT after about 5 h from 0.5 M $[Zn(bet)_2][NTf_2]_2$/AN solutions.

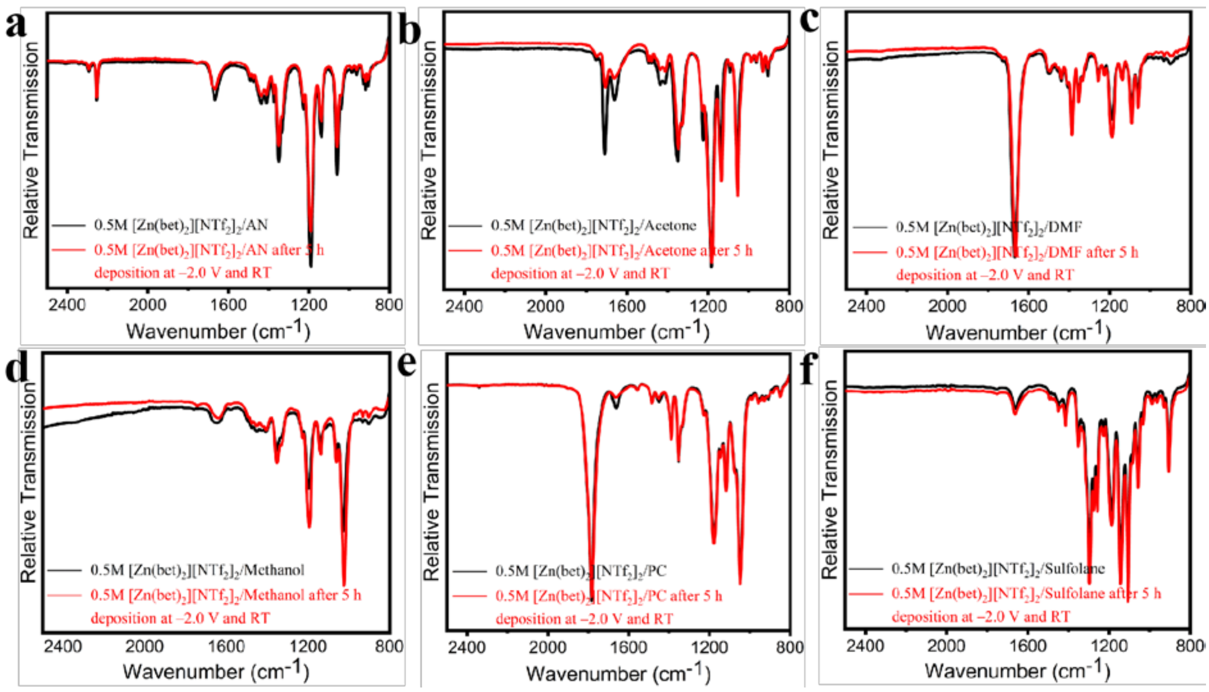

Figure S18. FT-IR spectra before (black) and after (red) 5 h Zn electrodeposition at –2.0 V and RT. **(a)** [Zn(bet)2][NTf2]2/AN solutions. **(b)** [Zn(bet)2][NTf2]2/acetone. **(c)** [Zn(bet)2][NTf2]2/DMF. **(d)** [Zn(bet)2][NTf2]2/methanol. **(e)** [Zn(bet)2][NTf2]2/PC. **(f)** [Zn(bet)2][NTf2]2/sulfolane.

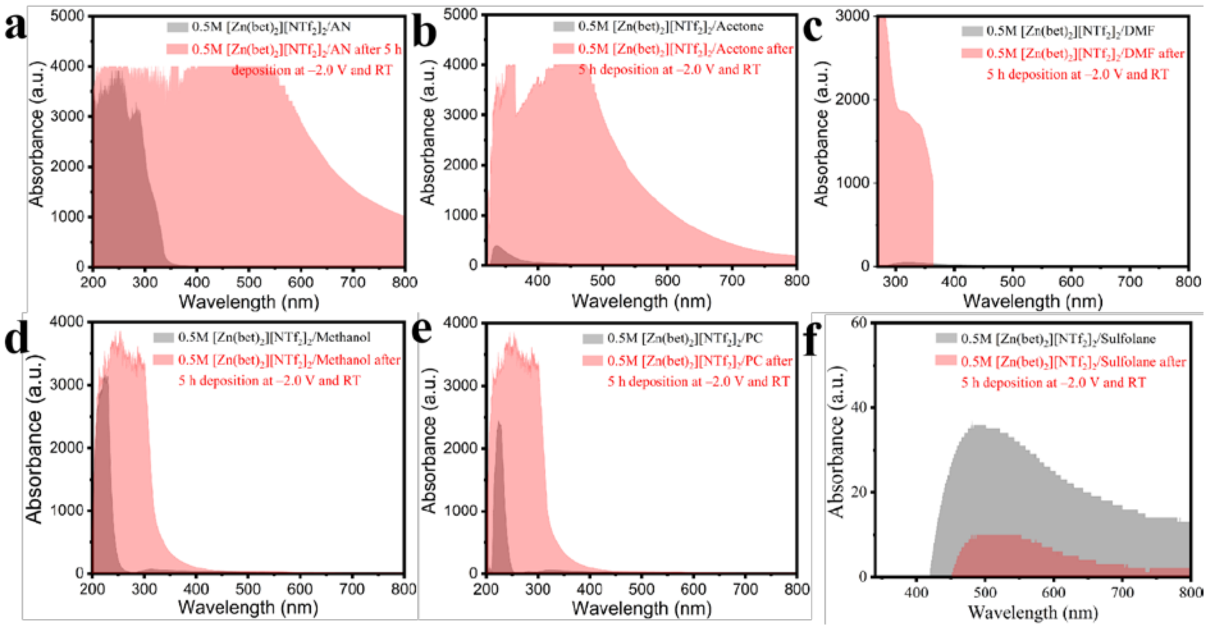

Figure S19. UV-Vis absorption spectra before and after 5 h Zn electrodeposition at –2.0 V and RT. **(a)** [Zn(bet)2][NTf2]2/AN solutions. **(b)** [Zn(bet)2][NTf2]2/acetone. **(c)** [Zn(bet)2][NTf2]2/DMF. **(d)** [Zn(bet)2][NTf2]2/methanol. **(e)** [Zn(bet)2][NTf2]2/PC. **(f)** [Zn(bet)2][NTf2]2/sulfolane.

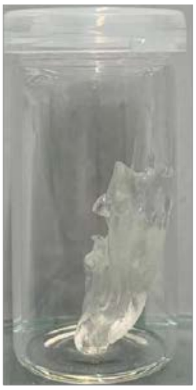

Figure S20. Photograph of $[Pb(bet)_2][NTf_2]_2$ at room temperature.

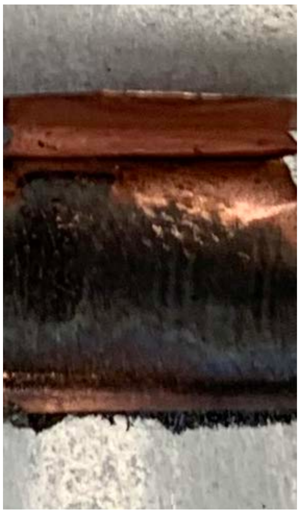

Figure S21. Photograph of electrodeposited Pb on Cu substrate at –1.17 V and RT from 0.5 M $[Pb(bet)_2][NTf_2]_2$/AN solutions.

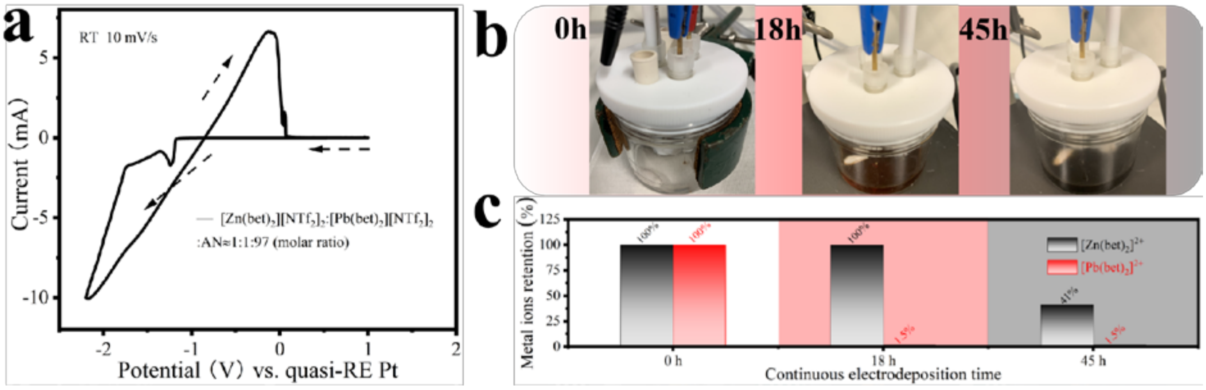

Figure S22. (a) CV of a mixture of $[Zn(bet)_2][NTf_2]_2$ and $[Pb(bet)_2][NTf_2]_2$ in AN ($n_{[Zn(bet)2][NTf2]2}$: $n_{[Pb(bet)2][NTf2]2}$: n_{AN} ≈ 1 : 1 : 97 or $m_{[Zn(bet)2][NTf2]2}$: $m_{[Pb(bet)2][NTf2]2}$: m_{AN} ≈ 1.078 g : 1.2567 g : 5 g) at a scan rate of 10 mV s^{-1} and RT. **(b)** Photographs of the bimetallic solution before and after 18 h at –1.40 V (Pb) and 27 h at –2.0 V (Zn) electrodeposition. **(c)** Metal complex retention with continuous electrodeposition (based on the weight of the electrodeposit metal).

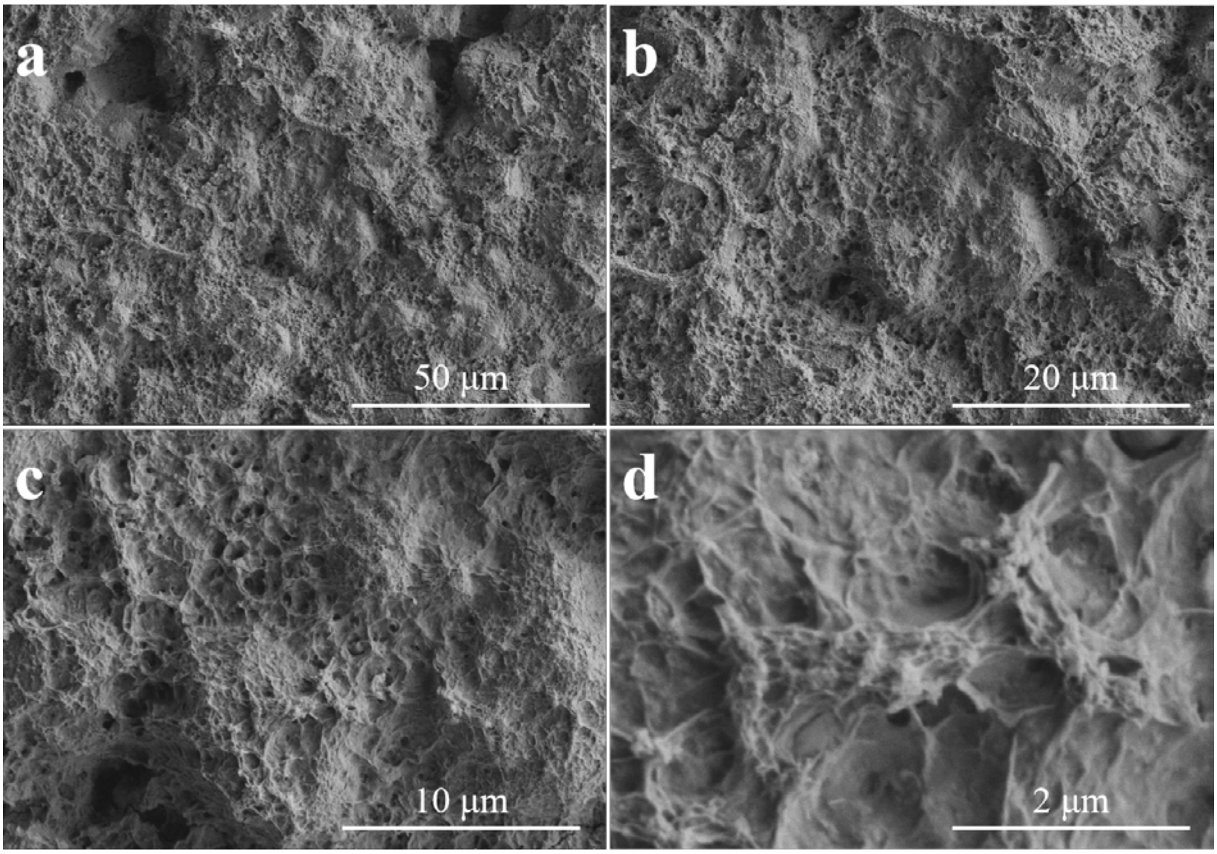

Figure S23. (a-d) SEM images (increasing magnification) of electrodeposited Pb on Cu foil substrate at –1.40 V and RT after about 18 h from $[Zn(bet)_2][NTf_2]_2$ and $[Pb(bet)_2][NTf_2]_2$ mixture in AN solutions ($n_{[Zn(bet)2][NTf2]2}$: $n_{[Pb(bet)2][NTf2]2}$: n_{AN} ≈ 2 : 1 : 97) solutions.

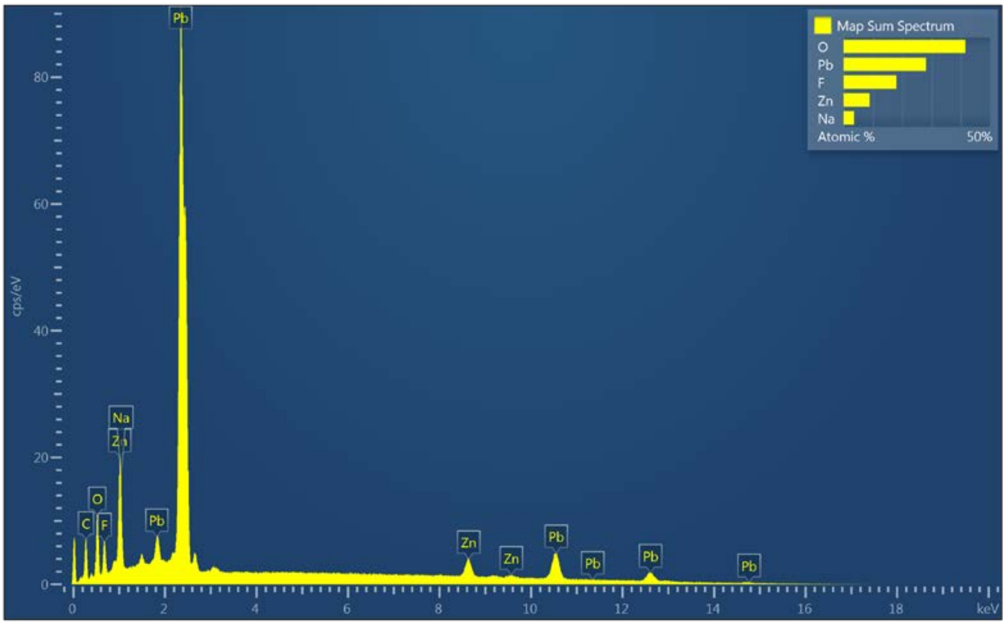

Figure S24. EDX analysis of electrodeposited Pb on Cu foil substrate at –1.40 V and RT after about 18 h from $[Zn(bet)_2][NTf_2]_2$ and $[Pb(bet)_2][NTf_2]_2$ mixture in AN solutions ($n_{[Zn(bet)2][NTf2]2}$: $n_{[Pb(bet)2][NTf2]2}$: n_{AN} ≈ 2 : 1 : 97) solutions.

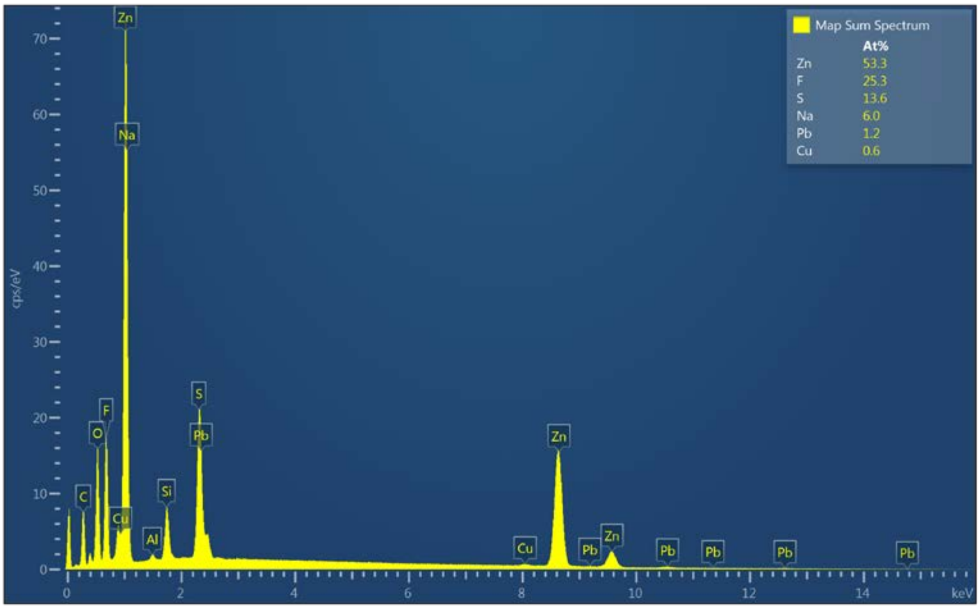

Figure S25. EDX analysis of electrodeposited Zn on Cu foil substrate at –2.0 V and RT after about 27 h from $[Zn(bet)_2][NTf_2]_2$ and $[Pb(bet)_2][NTf_2]_2$ mixture in AN solutions ($n_{[Zn(bet)2][NTf2]2}$: $n_{[Pb(bet)2][NTf2]2}$: n_{AN} ≈ 2 : 1 : 97) solutions.

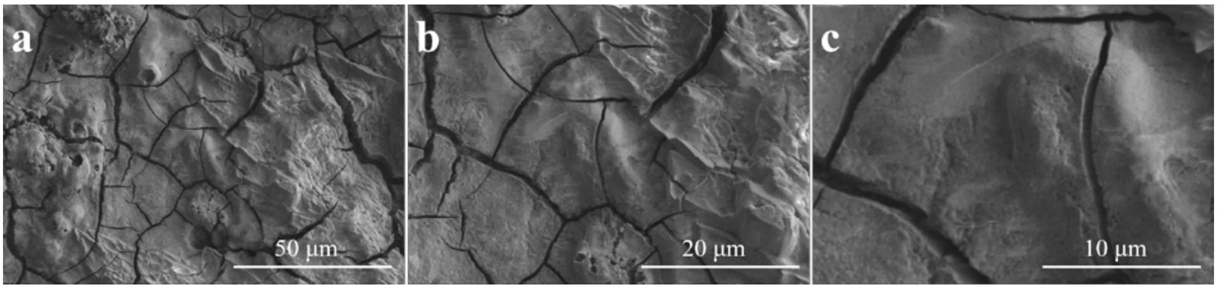

Figure S26. (a-c) SEM images (increasing magnification) of electrodeposited Zn on Cu foil substrate at –2.0 V and RT after about 27 h from $[Zn(bet)_2][NTf_2]_2$ and $[Pb(bet)_2][NTf_2]_2$ mixture in AN solutions ($n_{[Zn(bet)2][NTf2]2}$: $n_{[Pb(bet)2][NTf2]2}$: n_{AN} ≈ 2 : 1 : 97) solutions.

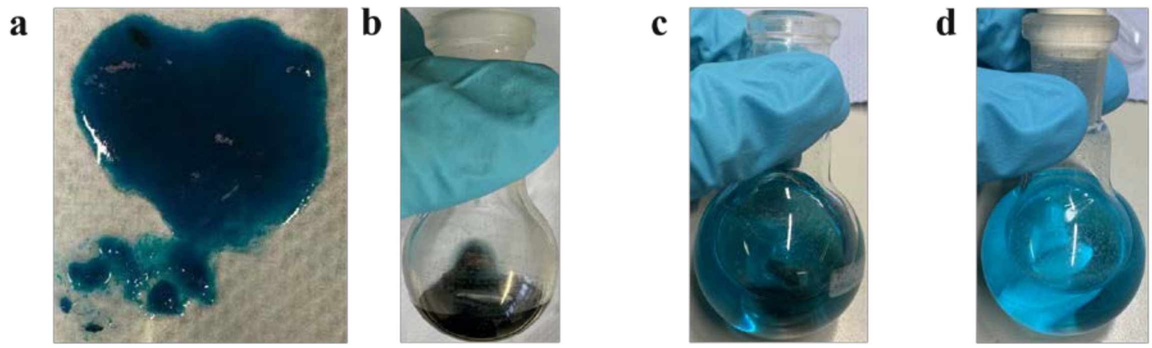

Figure S27. Photographs of **(a)** $[Cu_2(bet)_4(NTf_2)_2][NTf_2]_2$ synthesized from CuO, IL [Hbet][NTf_2] and additional 1.2 mg [Hbet]Cl (n_{CuO} : n_{IL} = 1 : 4) at RT. **(b)** $[Zn(bet)_2][NTf_2]_2$, $[Pb(bet)_2][NTf_2]_2$ and $[Cu_2(bet)_4(NTf_2)_2][NTf_2]_2$ mixture. **(c)** $[Zn(bet)_2][NTf_2]_2$, $[Pb(bet)_2][NTf_2]_2$ and $[Cu_2(bet)_4(NTf_2)_2][NTf_2]_2$ mixture dissolved in AN. **(d)** $[Zn(bet)_2][NTf_2]_2$, $[Pb(bet)_2][NTf_2]_2$ and $[Cu_2(bet)_4(NTf_2)_2][NTf_2]_2$ mixture dissolved in AN solutions after centrifugation.

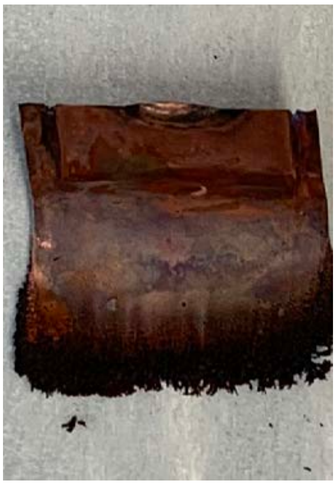

Figure S28. Photograph of electrodeposited Cu on Cu substrate at –1.17 V and RT from 0.5 M $[Cu_2(bet)_4(NTf_2)_2][NTf_2]_2$/AN solutions.

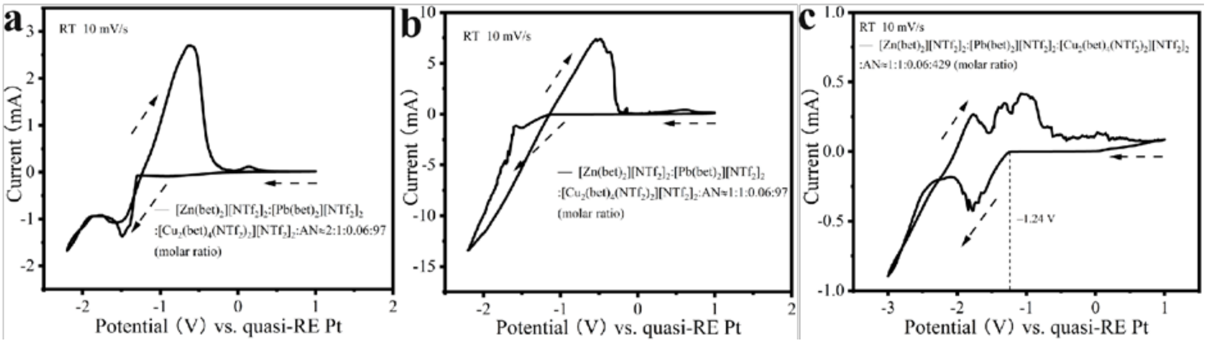

Figure S29. CVs of solutions of $[Zn(bet)_2][NTf_2]_2$, $[Pb(bet)_2][NTf_2]_2$ and $[Cu_2(bet)_4(NTf_2)_2][NTf_2]_2$ mixture in AN at a scan rate of 10 mV s^{-1} and RT. **(a)** $n_{[Zn(bet)2][NTf2]2}$: $n_{[Pb(bet)2][NTf2]2}$: $n_{[Cu2(bet)4(NTf2)2][NTf2]2}$: n_{AN} ≈ 2 : 1 : 0.06 : 97. **(b)** $n_{[Zn(bet)2][NTf2]2}$: $n_{[Pb(bet)2][NTf2]2}$: $n_{[Cu2(bet)4(NTf2)2][NTf2]2}$: n_{AN} ≈ 1 : 1 : 0.06 : 97 ($m_{[Zn(bet)2][NTf2]2}$: $m_{[Pb(bet)2][NTf2]2}$: $m_{[Cu2(bet)4(NTf2)2][NTf2]2}$: m_{AN} ≈ 1.078 g : 1.2567 g : 0.1415 g : 5 g). **(c)** $n_{[Zn(bet)2][NTf2]2}$: $n_{[Pb(bet)2][NTf2]2}$: $n_{[Cu2(bet)4(NTf2)2][NTf2]2}$: n_{AN} ≈ 1 : 1 : 0.06 : 429 ($m_{[Zn(bet)2][NTf2]2}$: $m_{[Pb(bet)2][NTf2]2}$: $m_{[Cu2(bet)4(NTf2)2][NTf2]2}$: m_{AN} ≈ 1.078 g : 1.2567 g : 0.1415 g : 22.1 g).

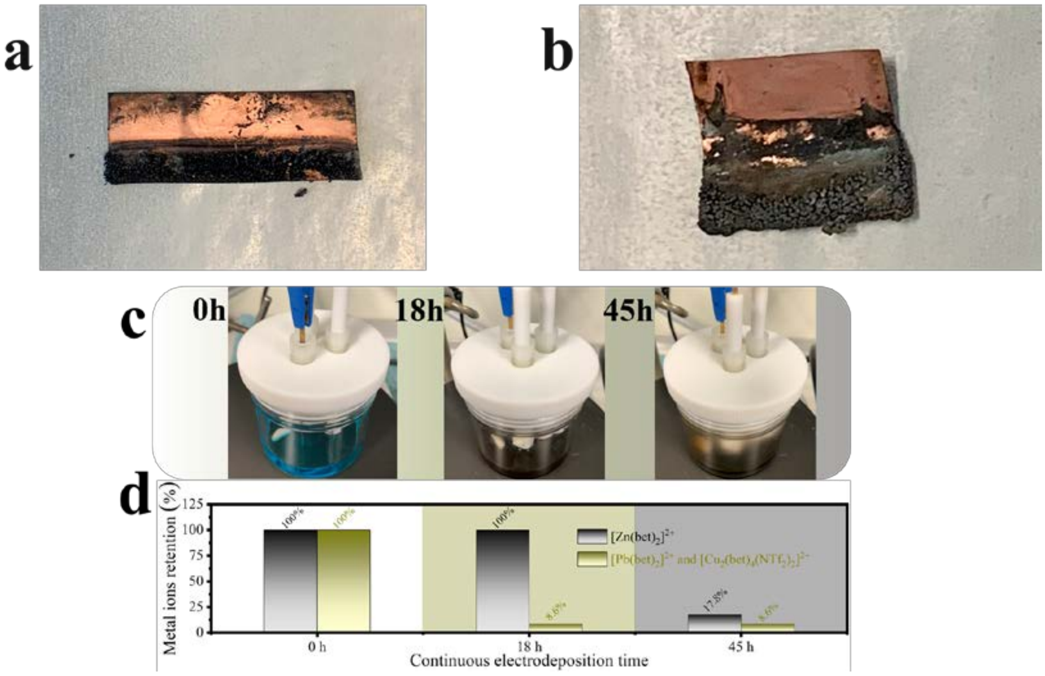

Figure S30. Photographs of **(a)** electrodeposited Co-ED Pb/Cu on Cu foil substrate at –1.40 V and RT after about 18 h from $[Zn(bet)_2][NTf_2]_2$, $[Pb(bet)_2][NTf_2]_2$ and $[Cu_2(bet)_4(NTf_2)_2][NTf_2]_2$ mixture in AN solutions ($n_{[Zn(bet)2][NTf2]2}$: $n_{[Pb(bet)2][NTf2]2}$: $n_{[Cu2(bet)4(NTf2)2][NTf2]2}$: n_{AN} ≈ 2 : 1 : 0.06 : 97). **(b)** Electrodeposited Zn on Cu foil substrate at –2.0 V and RT after about 27 h. **(c)** The photographs of primary three-mixture solutions and after 18 h at –1.40 V (Co-ED: Pb/Cu) and 27 h at –2.0 V (Zn) electrodeposition. **(d)** Metal complex retention with continuous electrodeposition (based on the weight of the electrodeposit metal).

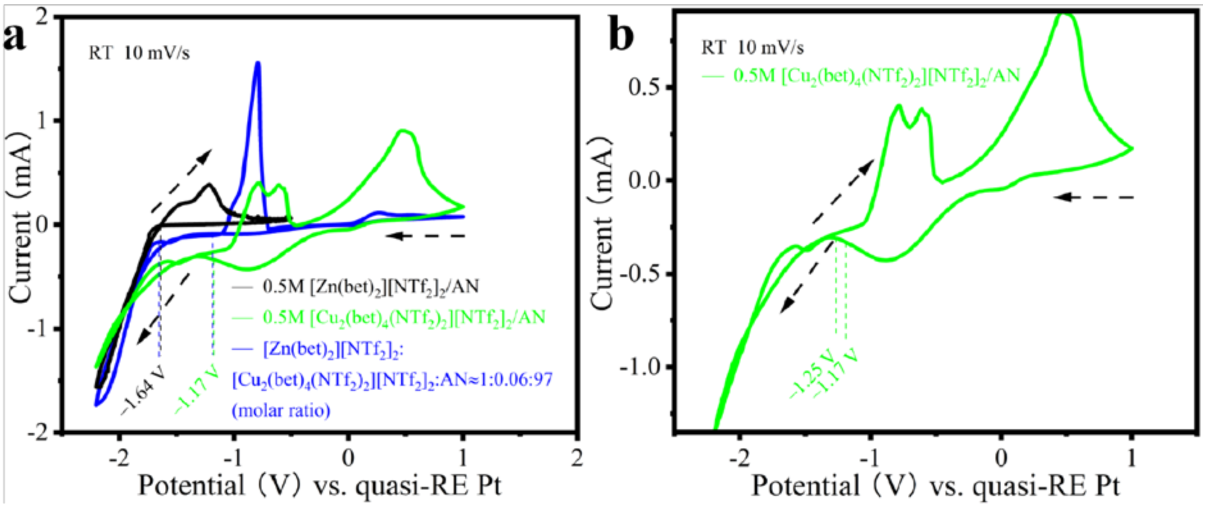

Figure S31. (a) CVs of AN solutions (0.5 M) of pure $[Zn(bet)_2][NTf_2]_2$, $[Cu_2(bet)_4(NTf_2)_2][NTf_2]_2$ and their mixture ($n_{[Zn(bet)2][NTf2]2}$: $n_{[Cu2(bet)4(NTf2)2][NTf2]2}$: n_{AN} ≈ 1 : 0.06 : 97 or $m_{[Zn(bet)2][NTf2]2}$: $m_{[Cu2(bet)4(NTf2)2][NTf2]2}$: m_{AN} ≈ 1.078 g : 0.1415 g : 5 g) at a scan rate of 10 mV s^{-1} and RT. **(b)** The enlarged green CV curve in the figure a.

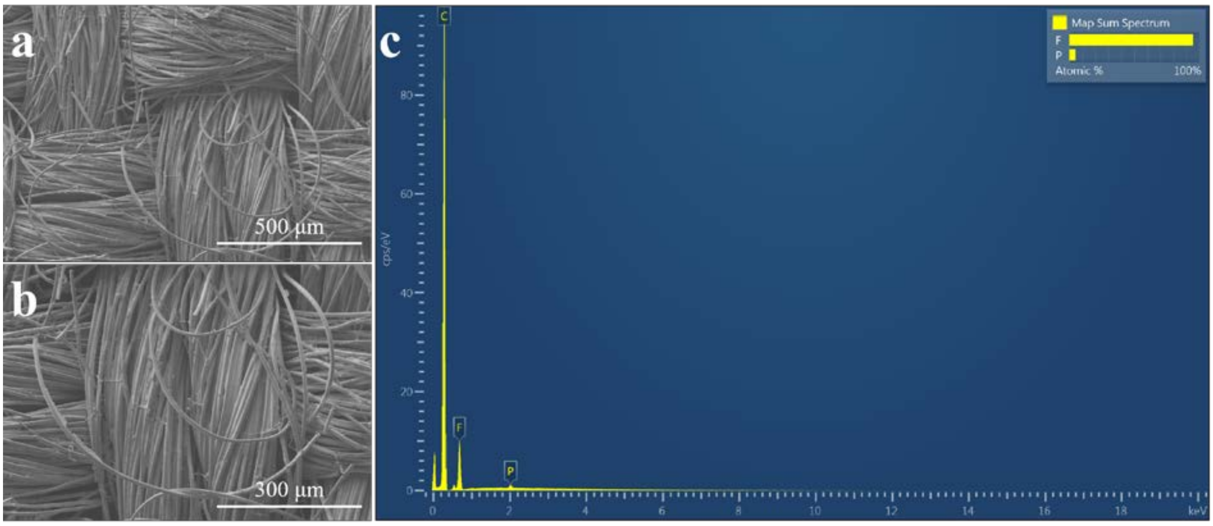

Figure S32. SEM images **(a-b)** and EDX analysis **(c)** of ^{3D}CF.

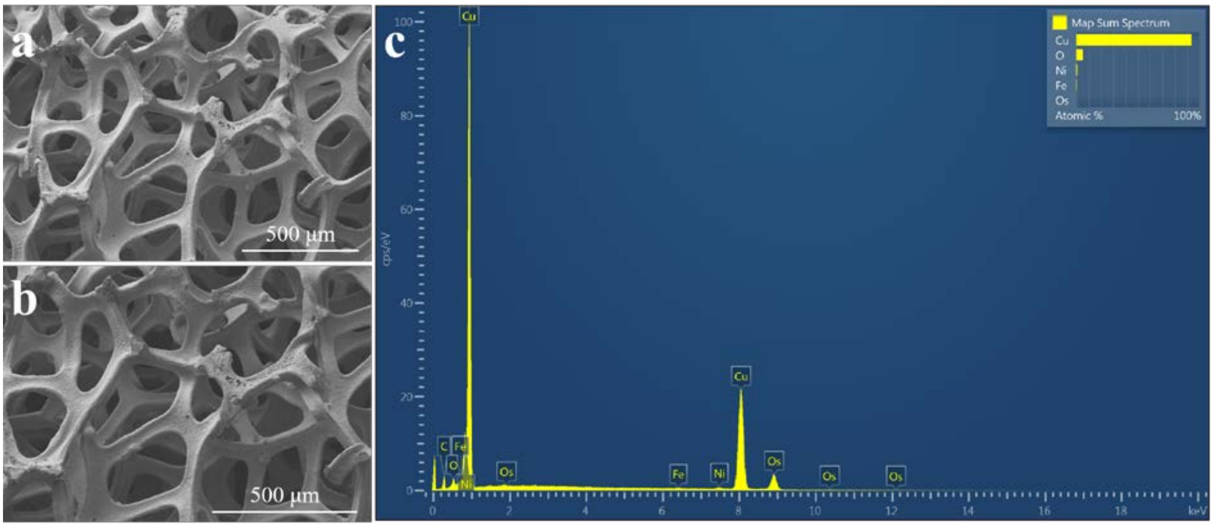

Figure S33. SEM images **(a-b)** and EDX analysis **(c)** of ^{3D}Cu.

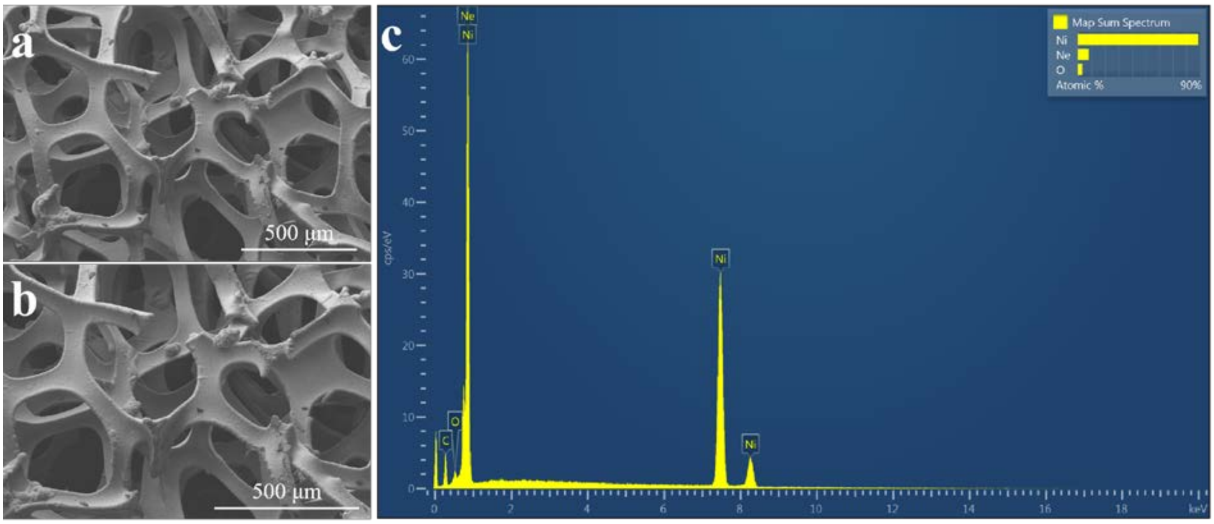

Figure S34. SEM images **(a-b)** and EDX analysis **(c)** of ^{3D}Ni.

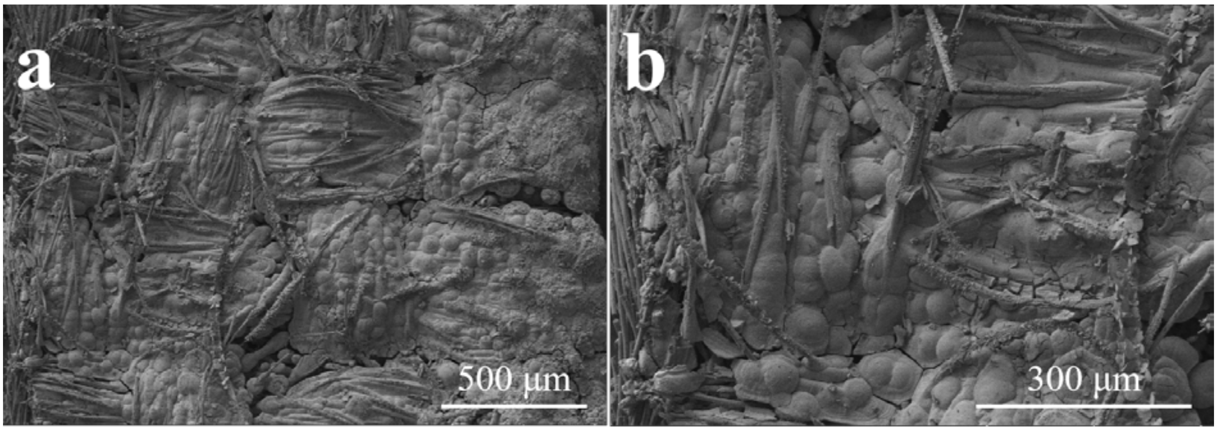

Figure S35. (a-b) SEM images of Zn/^{3D}CF electrodeposited at –2.0 V and RT from 0.5 M $[Zn(bet)_2][NTf_2]_2$/AN solutions.

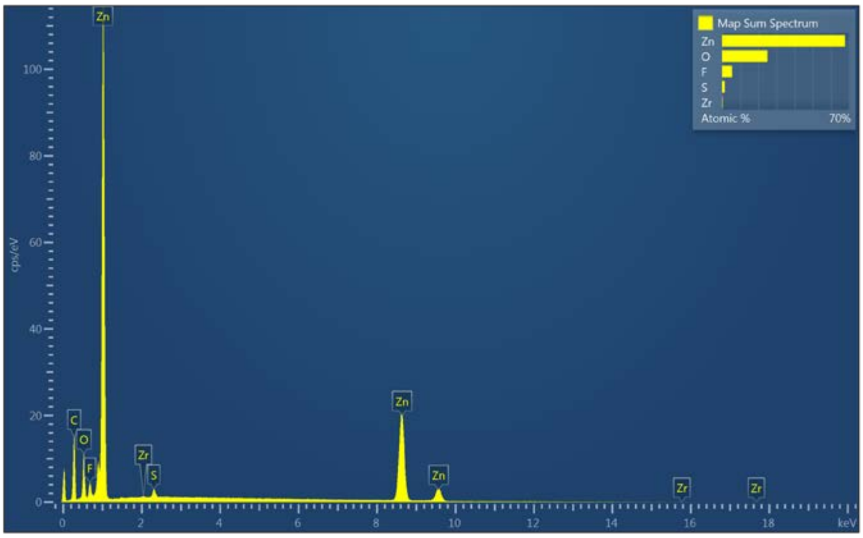

Figure S36. EDX analysis of Zn/^{3D}CF electrodeposited at –2.0 V and RT from 0.5 M $[Zn(bet)_2][NTf_2]_2$/AN solutions.

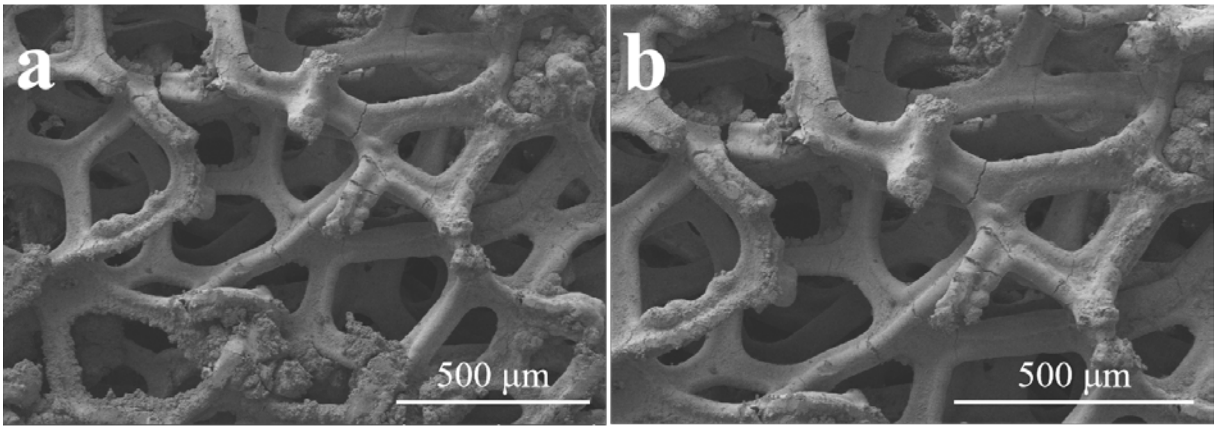

Figure S37. (a-b) SEM images of Zn/^{3D}Cu electrodeposited at –2.0 V and RT from 0.5 M [Zn(bet)$_2$][NTf$_2$]$_2$/AN solutions.

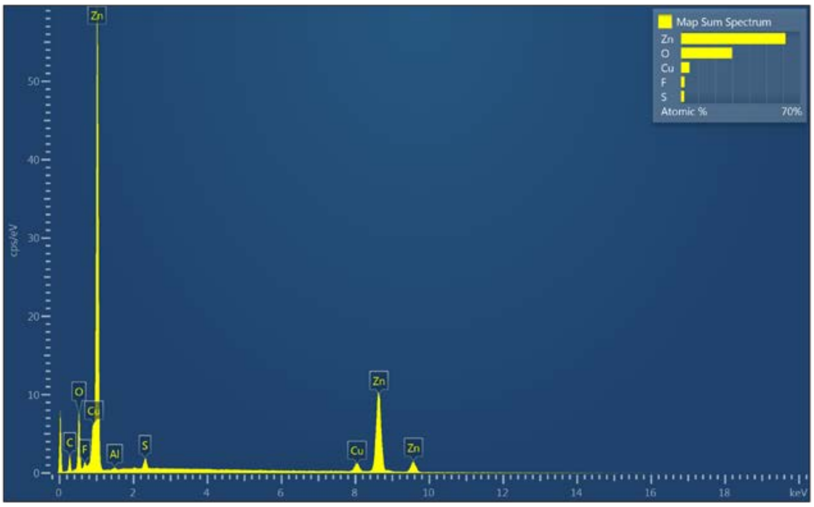

Figure S38. EDX analysis of Zn/^{3D}Cu electrodeposited at –2.0 V and RT from 0.5 M [Zn(bet)$_2$][NTf$_2$]$_2$/AN solutions.

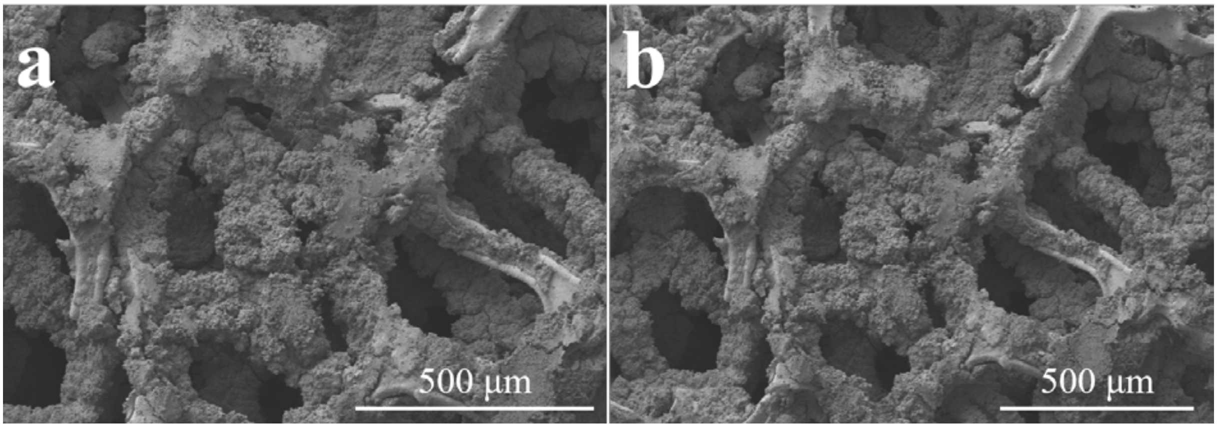

Figure S39. (a-b) SEM images of Zn/[3D]Ni electrodeposited at –2.0 V and RT from 0.5 M $[Zn(bet)_2][NTf_2]_2$/AN solutions.

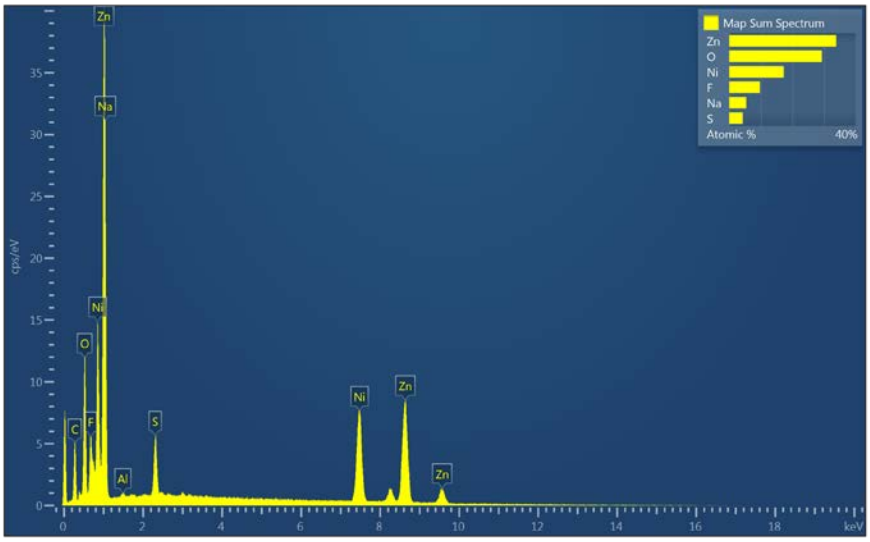

Figure S40. EDX analysis of Zn/[3D]Ni electrodeposited at –2.0 V and RT from 0.5 M $[Zn(bet)_2][NTf_2]_2$/AN solutions.

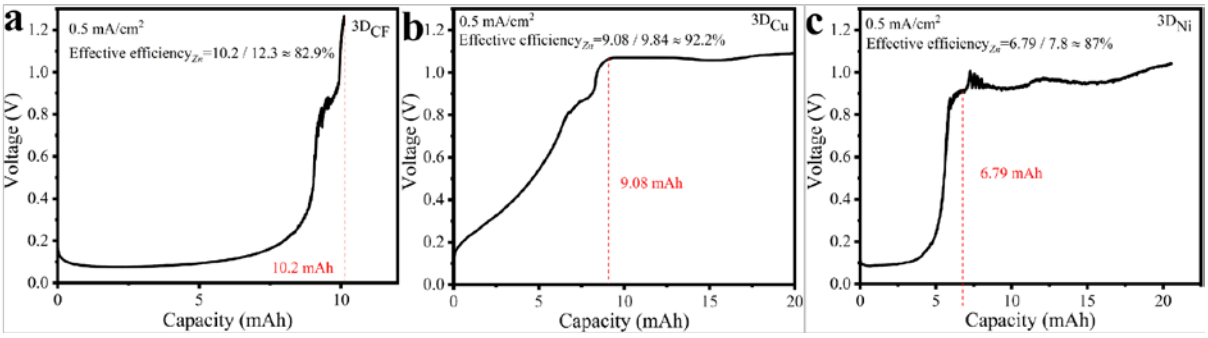

Figure S41. Galvanostatic charge curve of the Zn/^{3D}*M* anodes at 0.5 mA cm^{-2}. **(a)** Zn/^{3D}CF. **(b)** Zn/^{3D}Cu. **(c)** Zn/^{3D}Ni.

More promisingly, in a mixed electrolyte consisting of $LiPF_6$ and $Zn[NTf_2]_2$ in EMC, galvanostatic charge tests (Figure S41) showed an effective Zn deposition efficiency of about 92.2% on a Zn/^{3D}Cu anode. The explanation is that the Zn/^{3D}Cu anode (Figure 4o_2) provides a stable structure with a persistent labyrinth compared to the dense Zn/^{3D}CF (Figure 4k_2) and loose Zn/^{3D}Ni (Figure 4s_2), so that not only the electrolyte fully penetrates, but also the Zn does not detach, allowing more Zn to act electrochemically.

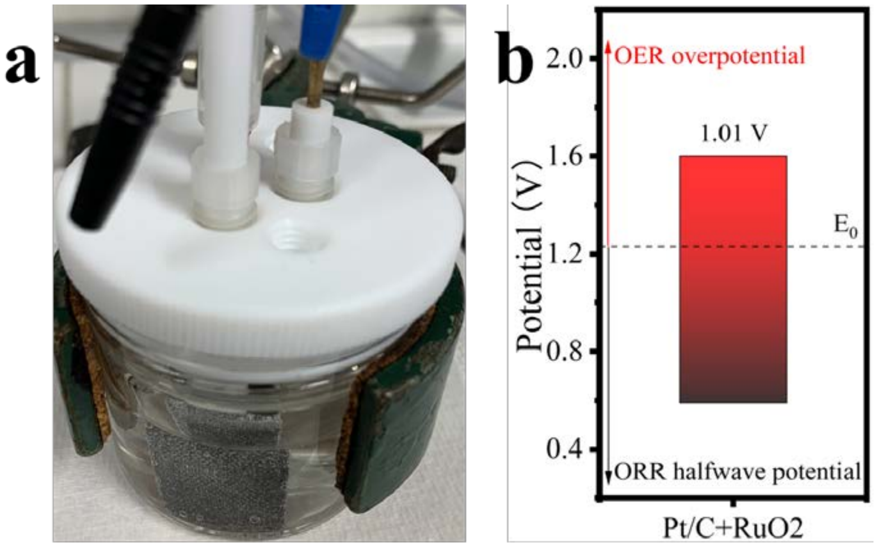

Figure S42. (a) Photograph of the OER electrocatalytic testing of Pt/C+RuO_2/CF air cathode. **(b)** The ORR and OER potential gap.

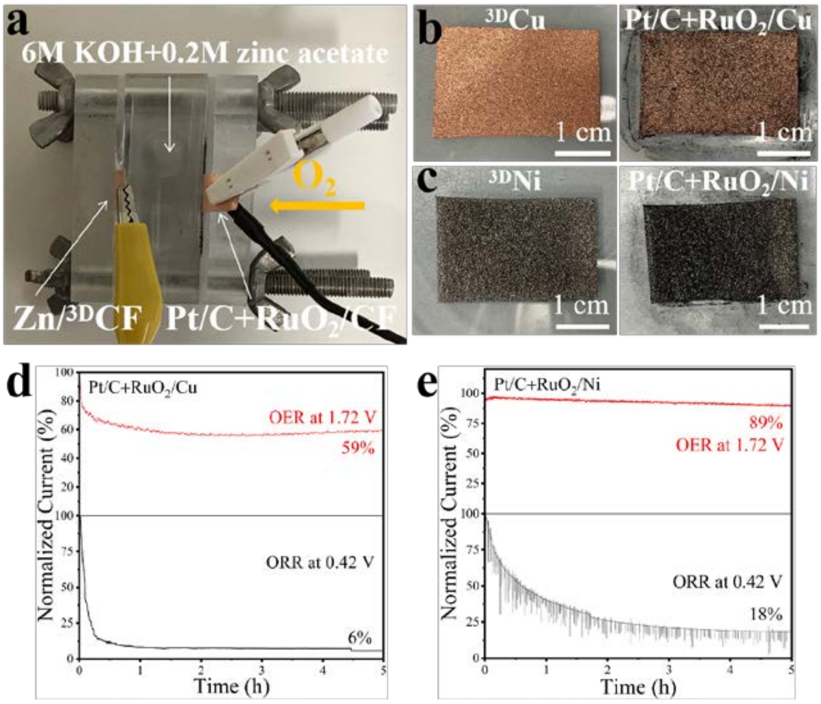

Figure S43. (a) The structure of the homemade ZABs using Zn/^{3D}CF anode and Pt/C+RuO_2/CF cathode. The optical photos of ^{3D}Cu **(b** left**)**, ^{3D}Ni **(c** left**)**, Pt/C+RuO_2/Cu **(b** right**)** and Pt/C+RuO_2/Ni **(c** right**)**. The ORR and OER polarization curves of Pt/C+RuO_2/Cu **(d)** or Pt/C+RuO_2/Ni **(e)**.

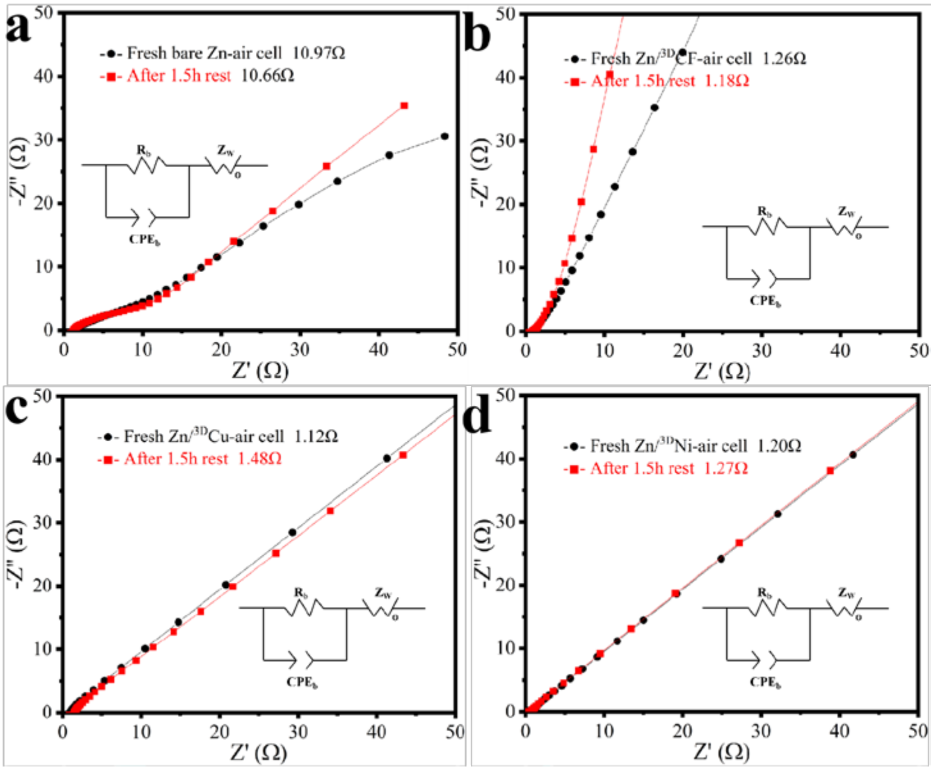

Figure S44. The EIS of the fresh and after 90 min rest aqueous rechargeable ZABs with Pt/C+RuO_2/CF air cathode and Zn/^{3D}CF **(b)**, Zn/^{3D}Cu **(c)**, Zn/^{3D}Ni **(d)** or comparative Zn foil **(a)** anodes (inset: the equivalent circuit for the simulation).

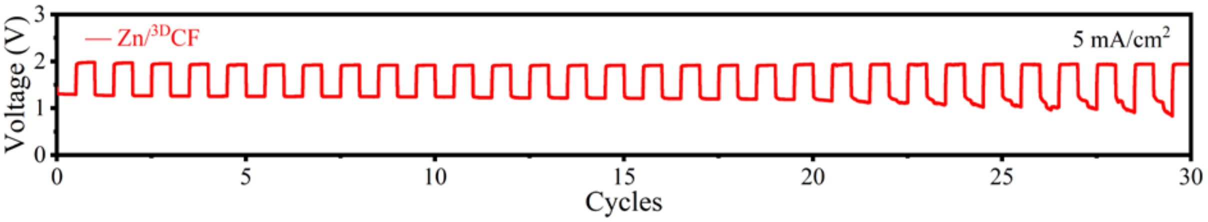

Figure S45. Galvanostatic discharge-charge cycling curves of aqueous rechargeable ZABs at 5.0 mA cm^{-2}, the charge/discharge depth: 600 s cycle^{-1}.

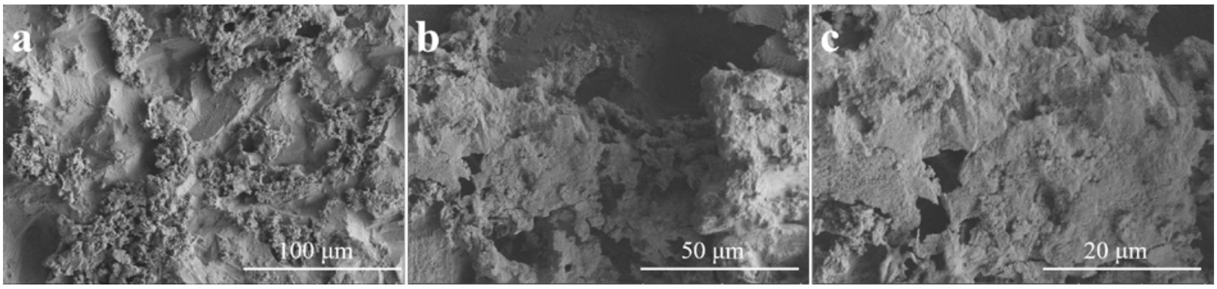

Figure S46. (a-c) SEM images (increasing magnification) of a cycled bare Zn anode.

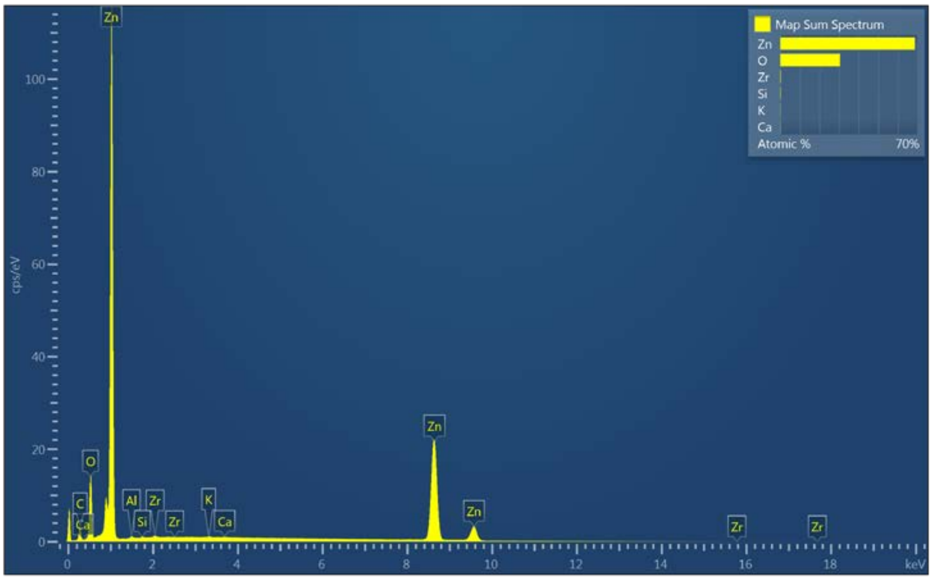

Figure S47. EDX analysis of a cycled bare Zn anode.

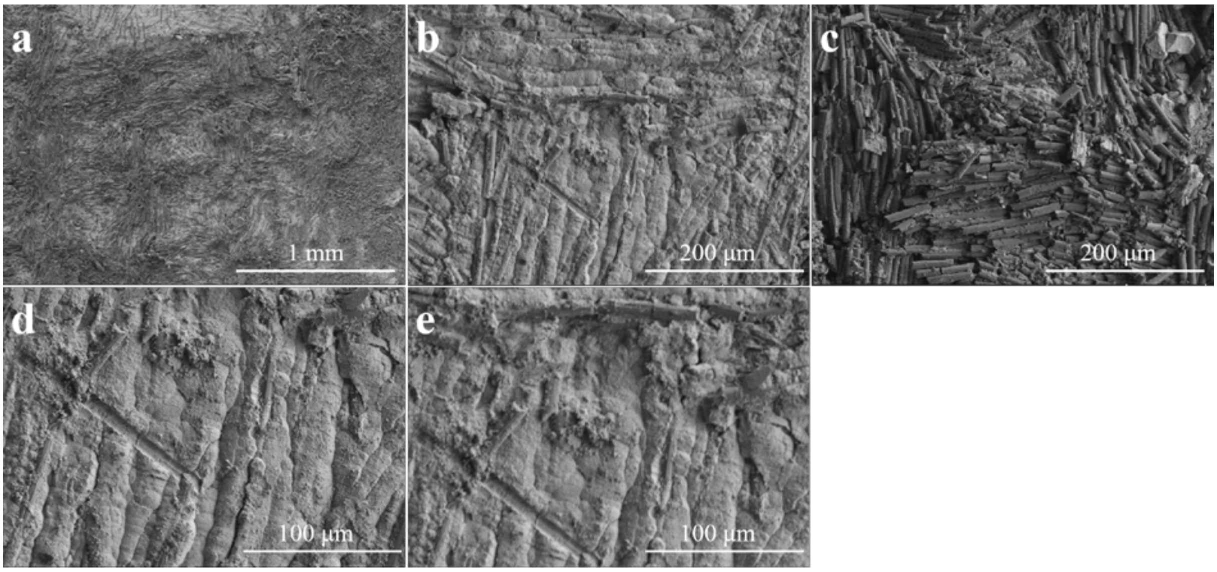

Figure S48. (a-e) SEM images (increasing magnification) of a cycled Zn/^{3D}CF anode.

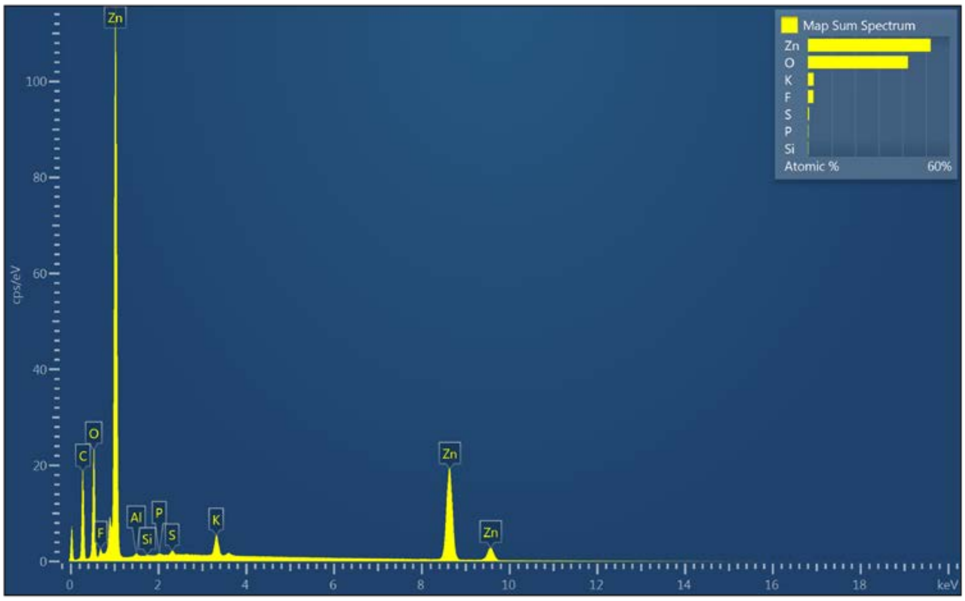

Figure S49. EDX analysis of a cycled Zn/^{3D}CF anode.

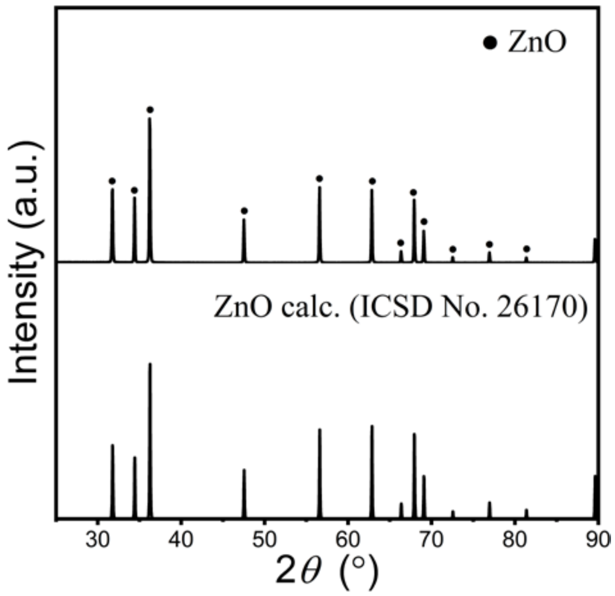

Figure S50. Comparison of the PXRD pattern of the ZnO used in this work with the reference.

Table S1. Comparison of the electrochemistry performance of various state-of-the-art ZABs.

Anode	Cathode	Open circuit potential / V	Power density / MW cm^{-2}	Reference
Zn plate	Co/Co_3O_4@PGS	1.45	118.3	[1]
Zn anode	C-MOF-C_2 900	1.46	105	[2]
Zn plate	NiO/CoN PINW	1.46	79.6	[3]
Zn plate	$CuCo_2O_4$/N-CNT	1.36	83.8	[4]
Zn plate	$ZnCo_2O_4$/N-CNT	1.47	82.3	[5]
Zn plate	CoPx@CNS	1.4	110	[6]
Zn plate	3DOM-Co@TiOxNy	1.466	110	[7]
Zn plate	Co_3O_{4-x} HoNPs@HPNC	1.459	94.1	[8]
Zn foil	Co_2FeO_4/NCNTs	1.43	90.68	[9]
Zn plate	Fe_{20}@N/HCSs	1.57	140.8	[10]
**Zn/^{3D}CF**	**Pt/C+RuO_2/CF**	**1.631 (initial)**<br>**1.48 (after 90 min rest)**	**96.03**	**this work**

References

[1] Y. Jiang, Y. P. Deng, J. Fu, D. U. Lee, R. Liang, Z. P. Cano, Y. Liu, Z. Bai, S. Hwang, L. Yang, D. Su, W. Chu, Z. Chen, *Adv. Energy Mater.* **2018**, *8*, 1.

[2] M. Zhang, Q. Dai, H. Zheng, M. Chen, L. Dai, *Adv. Mater.* **2018**, *30*, 1.

[3] J. Yin, Y. Li, F. Lv, Q. Fan, Y. Q. Zhao, Q. Zhang, W. Wang, F. Cheng, P. Xi, S. Guo, *ACS Nano* **2017**, *11*, 2275.

[4] H. Cheng, M. L. Li, C. Y. Su, N. Li, Z. Q. Liu, *Adv. Funct. Mater.* **2017**, *27*, 1.

[5] Z. Q. Liu, H. Cheng, N. Li, T. Y. Ma, Y. Z. Su, *Adv. Mater.* **2016**, *28*, 3777.

[6] C. C. Hou, L. Zou, Y. Wang, Q. Xu, *Angew. Chem. Int. Ed.* **2020**, *59*, 21360.

[7] G. Liu, J. Li, J. Fu, G. Jiang, G. Lui, D. Luo, Y. P. Deng, J. Zhang, Z. P. Cano, A. Yu, D. Su, Z. Bai, L. Yang, Z. Chen, *Adv. Mater.* **2019**, *31*, 1.

[8] D. Ji, L. Fan, L. Tao, Y. Sun, M. Li, G. Yang, T. Q. Tran, S. Ramakrishna, S. Guo, *Angew. Chem. Int. Ed.* **2019**, *58*, 13840.

[9] X. T. Wang, T. Ouyang, L. Wang, J. H. Zhong, T. Ma, Z. Q. Liu, *Angew. Chem. Int. Ed.* **2019**, *58*, 13291.

[10] B. Wang, Y. Ye, L. Xu, Y. Quan, W. Wei, W. Zhu, H. Li, J. Xia, *Adv. Funct. Mater.* **2020**, *2005834*, 1.
